# Genetic analysis of the *Drosophila* ESCRT-III complex protein, VPS24, reveals a novel function in lysosome homeostasis

**DOI:** 10.1371/journal.pone.0251184

**Published:** 2021-05-06

**Authors:** Jonathan R. Florian, Samuel J. DeMatte, Devon M. Sweeder, Richard W. Ordway, Fumiko Kawasaki

**Affiliations:** Department of Biology and Center for Cellular Dynamics, The Pennsylvania State University, University Park, PA, United States of America; EPFL, SWITZERLAND

## Abstract

The ESCRT pathway is evolutionarily conserved across eukaryotes and plays key roles in a variety of membrane remodeling processes. A new *Drosophila* mutant recovered in our forward genetic screens for synaptic transmission mutants mapped to the *vps24* gene encoding a subunit of the ESCRT-III complex. Molecular characterization indicated a loss of VPS24 function, however the mutant is viable and thus loss of VPS24 may be studied in a developed multicellular organism. The mutant exhibits deficits in locomotion and lifespan and, notably, these phenotypes are rescued by neuronal expression of wild-type VPS24. At the cellular level, neuronal and muscle cells exhibit marked expansion of a ubiquitin-positive lysosomal compartment, as well as accumulation of autophagic intermediates, and these phenotypes are rescued cell-autonomously. Moreover, VPS24 expression in glia suppressed the mutant phenotype in muscle, indicating a cell-nonautonomous function for VPS24 in protective intercellular signaling. Ultrastructural analysis of neurons and muscle indicated marked accumulation of the lysosomal compartment in the *vps24* mutant. In the neuronal cell body, this included characteristic lysosomal structures associated with an expansive membrane compartment with a striking tubular network morphology. These findings further define the *in vivo* roles of VPS24 and the ESCRT pathway in lysosome homeostasis and their potential contributions to neurodegenerative diseases characterized by defective ESCRT or lysosome function.

## Introduction

The ESCRT (Endosomal Sorting Complexes Required for Transport) pathway participates in a wide range of cellular processes in which membranes are remodeled to compartmentalize proteins and organelles [1–4]. Of particular relevance to the present study, these include a key role in maintaining cellular homeostasis by mediating lysosomal degradation of proteins and organelles through endosomal and autophagic pathways [5, 6]. Moreover, ESCRT-mediated formation of extracellular vesicles has been implicated in intercellular signaling [7].

Membrane remodeling through the ESCRT pathway typically involves budding of membranes away from the cytoplasm [4, 8]. This is carried out by four protein complexes: ESCRT-0, ESCRT-I, ESCRT-II and ESCRT-III, as well as the VPS4 ATPase [9]. These mechanisms are conserved among eukaryotes and their *in vivo* roles have been studied through genetic analysis in several model systems, including *Drosophila* [10–16]. Recent work indicates that the ESCRT pathway participates in membrane remodeling in different ways. In addition to membrane budding away from cytoplasm, the ESCRT machinery can mediate remodeling with the opposite topology, including peroxisome biosynthesis and endosome recycling through tubular intermediates [17–19]. Although these mechanisms remain incompletely understood, they suggest an even broader range of potential functions for the ESCRT pathway.

In the present study, we have generated a new mutant of the ESCRT component, VPS24, that has revealed novel aspects of ESCRT function. VPS24 is orthologous to the mammalian ESCRT-III component, CHMP3, which is one of four core ESCRT-III components involved in membrane remodeling, along with CHMP6, CHMP4 and CHMP2 [20, 21]. These ESCRT-III proteins assemble into filaments and work with VPS4 at a late step to mediate membrane deformation and fission [22–24]. Mutations in CHMP2 have been implicated in the neurodegenerative diseases, Frontotemporal Dementia (FTD) [25] and Amyotrophic Lateral Sclerosis (ALS) [26]. Previous studies in *Drosophila* have examined three of the core ESCRT-III components, VPS20 (CHMP6), VPS32/SHRUB (CHMP4) and VPS2 (CHMP2), and shown they are essential for viability [10, 14]. Genetic analysis of the fourth core ESCRT-III component, VPS24, is addressed in the present study. Although founding work in yeast has defined a critical function for VPS24 in formation of intraluminal vesicles in the endosome, referred to as multivesicular bodies (MVB) [20], VPS24/CHMP3 appears to play an accessory role in mammalian cells [27, 28]. Despite progress in establishing the structure and function of the ESCRT pathway and ESCRT-III complex, additional genetic analysis is needed to further define their *in vivo* contributions to membrane remodeling in multicellular organisms.

## Results

### A forward genetic screen and recovery of a *vps24* mutant

**Fig 1:**
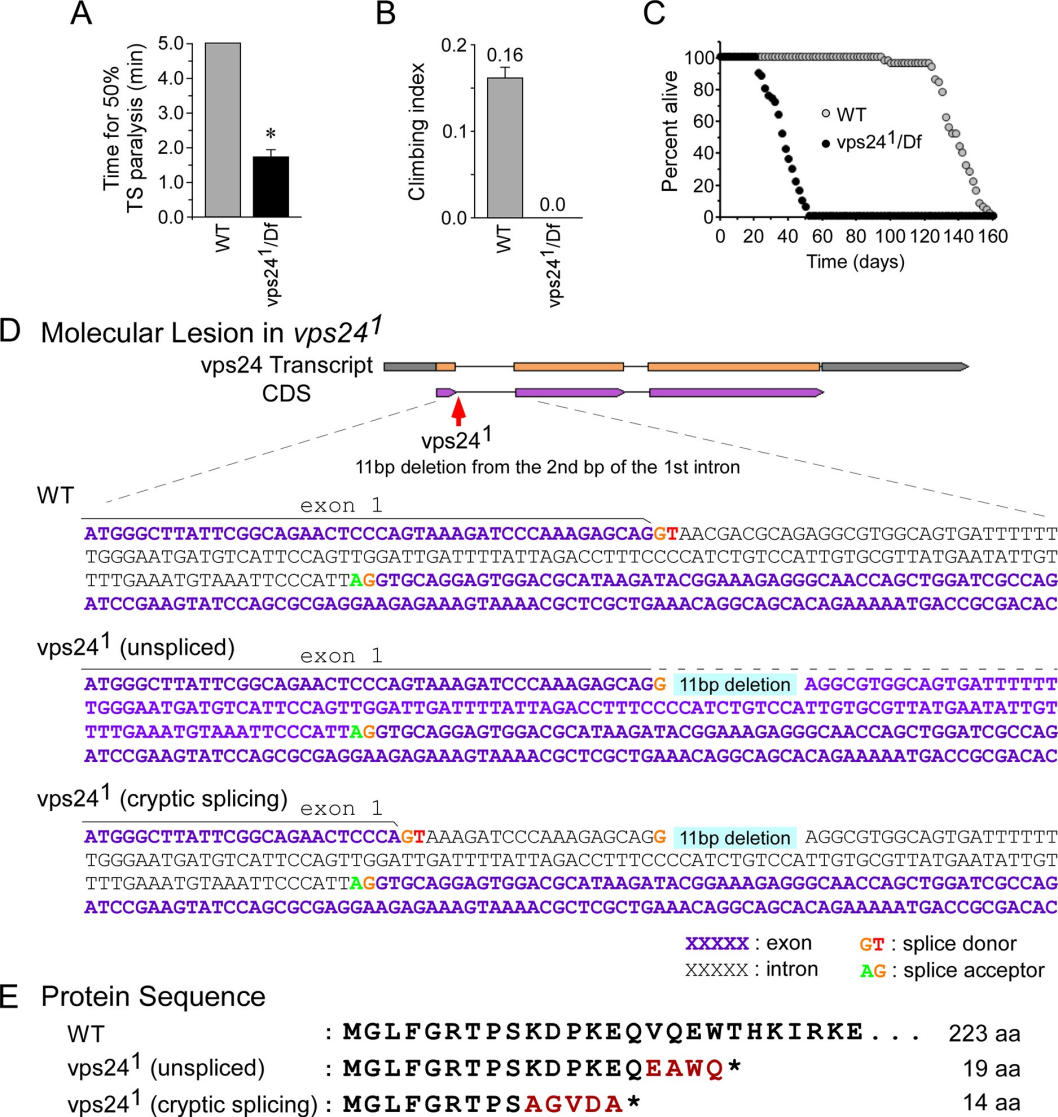
A new mutant of *vps24*, *vps24*^*1*^. **(A-C)** The *vps24* mutant exhibits locomotor and lifespan deficits. **(A)** *vps24*^*1*^ */Df(3R)Exel6140* flies (vps24^1^ /Df) exhibited rapid temperature sensitive (TS) paralysis at 38°C, whereas wild-type flies (WT) did not. Tests were truncated at 5 min if 50% TS paralysis had not occurred. **(B)** Climbing tests, carried out at a room temperature (RT) of 22–24°C, indicated no detectable climbing ability in the *vps24* mutant. The climbing tests were truncated at 2 min if 50% climbing had not occurred and zero was given for the climbing index (see Materials and Methods). **(C)** Loss of the VPS24 function reduced lifespan with respect to WT. 7d old female flies raised at 20°C were examined in these and the following behavioral studies. Here and in subsequent figures, data points represent the mean ± SEM and asterisks mark significant differences from control values (*P* = 0.05). **(D, E)** *vps24*^*1*^ mutation. **(D)** In *vps24*^*1*^, an 11 bp deletion occurs at the beginning of the 1st intron and removes its splice donor signal. This disrupts splicing of the 1st intron in two ways: complete failure of splicing to remove the first intron (unspliced) or use of a cryptic splice donor site contained within the WT Exon 1 (see text). The broken line above the vps24^1^ (unspliced) sequence represents continuation of Exon 1. **(E)** Each type of aberrant *vps24* transcript (unspliced or cryptically spliced) encodes a drastically truncated polypeptide composed of the first 15 or 9 amino acids of VPS24 (full length is 223) followed by several amino acids of non-VPS24 protein sequence (in red). *, stop codon.

Our previous work has involved genetic analysis of temperature-sensitive (TS) paralytic mutants to examine the molecular mechanisms of chemical synaptic transmission [29–34]. As an extension of this approach, the current study initiated a forward genetic screen for TS paralytic alleles of candidate genes implicated in synaptic function, including *complexin* and *sec1*/*Rop*. The screen incorporated deficiencies (deletions) for these genes with the goal of recovering recessive TS paralytic mutations on the basis of non-complementation. A new mutant recovered in this screen, 748 (*vps24*^*1*^), exhibited a recessive TS paralytic phenotype (Fig 1A, S1A and S1B Fig). However, this mutant was not strictly conditional in that it exhibited clear phenotypes at permissive temperature, including a significant decline in climbing performance at 7 days of age (Fig 1B) and a marked reduction in lifespan to a range of approximately 3–8 weeks (Fig 1C). The 748 mutation mapped to the deficiency, *Df(3R)Exel6140*, and complementation testing using existing mutations within this deficiency revealed that a P element allele of *vps24* failed to compliment the 748 mutant phenotype (S1C–S1E Fig). Thus, mutant 748 is allelic to the *vps24* gene, which encodes a subunit of ESCRT-III complex, and is designated *vps24*^*1*^.

### Molecular characterization of the *vps24*^*1*^ mutant

Sequence analysis of genomic DNA from the *vps24*^*1*^ mutant identified a molecular lesion in the *vps24* gene. An 11bp deletion from the 2nd bp of the 1st intron disrupts the splice donor sequence for this intron (Fig 1D). To examine whether this mutation alters splicing of *vps24* transcripts, PCR and sequence analysis of cDNAs was carried out. cDNA samples generated from whole-fly RNA of wild‐type (WT) and *vps24*^*1*^ mutant flies were analyzed by PCR using primers flanking the 1st intron. In the case of WT, we observed major and minor PCR products corresponding to spliced and unspliced *vps24* transcripts, respectively (S2A Fig). In contrast, the *vps24*^*1*^ mutant exhibited a distinct pattern of PCR products indicating an apparent increase in the level of unspliced transcript as well as a reduced size of both spliced and unspliced transcripts with respect to wild type (S2A Fig and caption). Sequence analysis of PCR products from the *vps24* mutant revealed two aberrant transcripts. One contains the unspliced 121bp intronic sequence between Exons 1 and 2 (Fig 1D, *vps24*^*1*^ unspliced). This creates a frame shift and is predicted to produce a truncated form of the protein including the first 15 amino acids followed by 4 amino acids of non-VPS24 sequence (Fig 1E). The second aberrant transcript contains a 20bp deletion with respect to the WT resulting from use of a cryptic splice site located 20bp upstream of the 1st intron (Fig 1D, *vps24*^*1*^ cryptic splicing). This is predicted to produce a truncated protein composed of the first 9 amino acids of VPS24 followed by 5 amino acids of non-VPS24 sequence (Fig 1E). These results indicate that the *vps24*^*1*^ mutant is likely to produce a complete loss of VPS24 function, however we cannot rule out that an undetectable transcript expresses a low level of functional protein. Finally, in the P element insertional allele of *vps24* which failed to complement *vps24*^*1*^, *vps24* transcript levels were reduced by more than 50% relative to *vps24*^*1*^ or WT (S2B Fig).

### Transformation rescue indicates a neuronal requirement for VPS24

**Fig 2:**
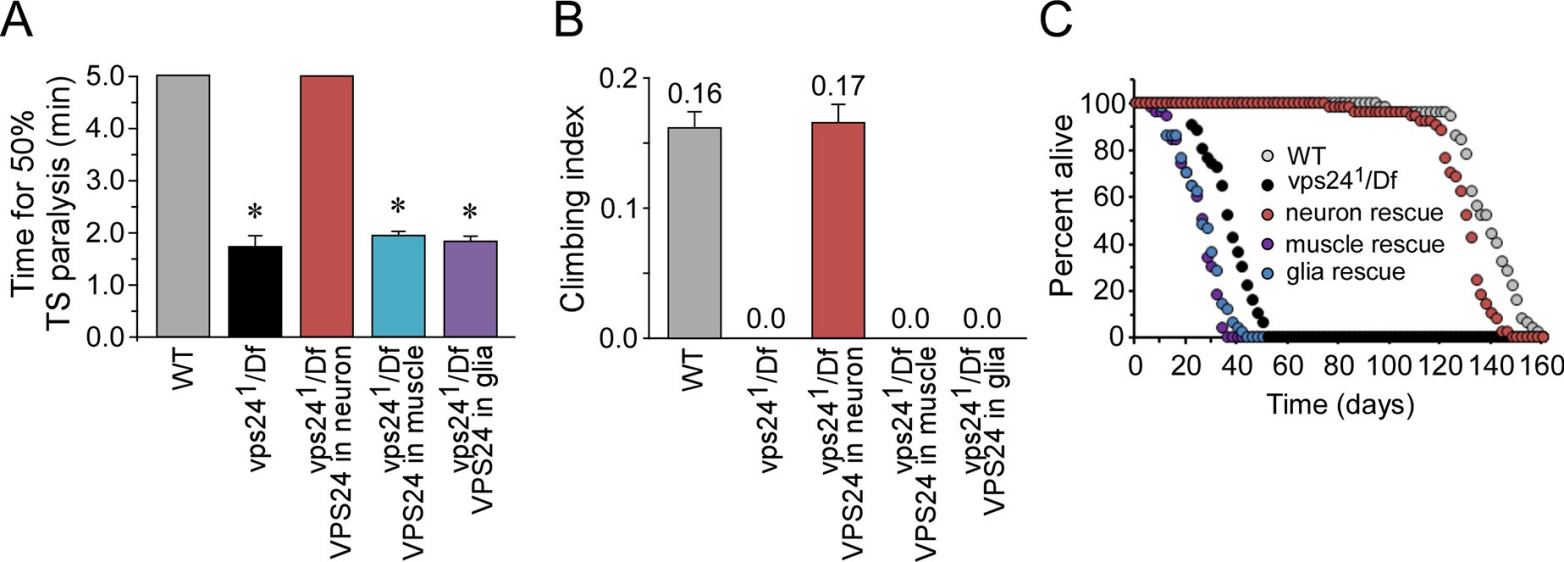
Neuronal expression of the wild-type VPS24 protein rescues the *vps24*^*1*^ TS paralytic phenotype as well as locomotor and lifespan deficits. **(A)** The TS paralytic phenotype of the *vps24* mutant was rescued by expression of the wild-type EGFP-VPS24 in neurons but not in muscle or glia. The GAL4 drivers for neuronal, muscle and glial expression were Appl-GAL4, Mhc-GAL4 and Repo-Gal4 respectively. **(B)** Climbing tests, carried out at a room temperature (RT) of 22–24°C, indicated no detectable climbing ability in the *vps24* mutant. The climbing tests were truncated at 2 min if 50% climbing had not occurred and zero was given for the climbing index. **(C)** Loss of the VPS24 function reduced lifespan with respect to WT when raised at a permissive temperature of 20°C. These mutant phenotypes were rescued by expression of wild-type EGFP-VPS24 in neurons but not in muscle or glia. The same GAL4 drivers were used for subsequent cell-type specific expression studies.

To confirm that the *vps24*^*1*^ behavioral phenotypes result from loss of VPS24 function, and to further characterize the requirement for VPS24, transformation rescue experiments were performed. These studies used the GAL4-UAS system which permits spatial and temporal control of transgene expression [35]. A UAS transgene and corresponding transgenic lines were generated to express wild-type VPS24 fused with green fluorescent protein (GFP) at its N-terminus. Notably, all the *vps24*^*1*^ behavioral phenotypes are rescued by transgenic expression of wild-type VPS24 in neurons, but not in glia or muscle, indicating a primary requirement for VPS24 in neurons (Fig 2). The nonconditional deficits in neuronal function at permissive temperature are consistent with the molecular lesions in the *vps24*^*1*^ and P element alleles and suggest the TS paralytic phenotype results from further compromise of neuronal mechanisms at elevated temperatures. The preceding findings reveal that VPS24 function in neurons is critical for basic motor function and viability, consistent with previous studies implicating ESCRT-III components in neurodegenerative disease [25, 26, 36, 37].

Since the initial genetic screen was for synaptic transmission mutants, and our results indicated a key role for VPS24 in neurons, a secondary screen was conducted to determine whether *vps24*^*1*^ exhibits a defect in synaptic function. Electrophysiological studies were performed at adult neuromuscular synapses of the flight motor [34, 38]. Voltage clamp recordings of excitatory postsynaptic currents (EPSCs) were carried out at synapses of the dorsal longitudinal flight muscles (DLMs) at both permissive and elevated temperatures [33, 34]. DLM neuromuscular synapse function was normal in *vps24*^*1*^ with respect to the amplitude and waveform of individual EPSCs as well as short-term plasticity (S3 Fig) and studies of synaptic transmission were not further pursued in this mutant.

### Cellular phenotypes in the *vps24*^*1*^ mutant

**Fig 3:**
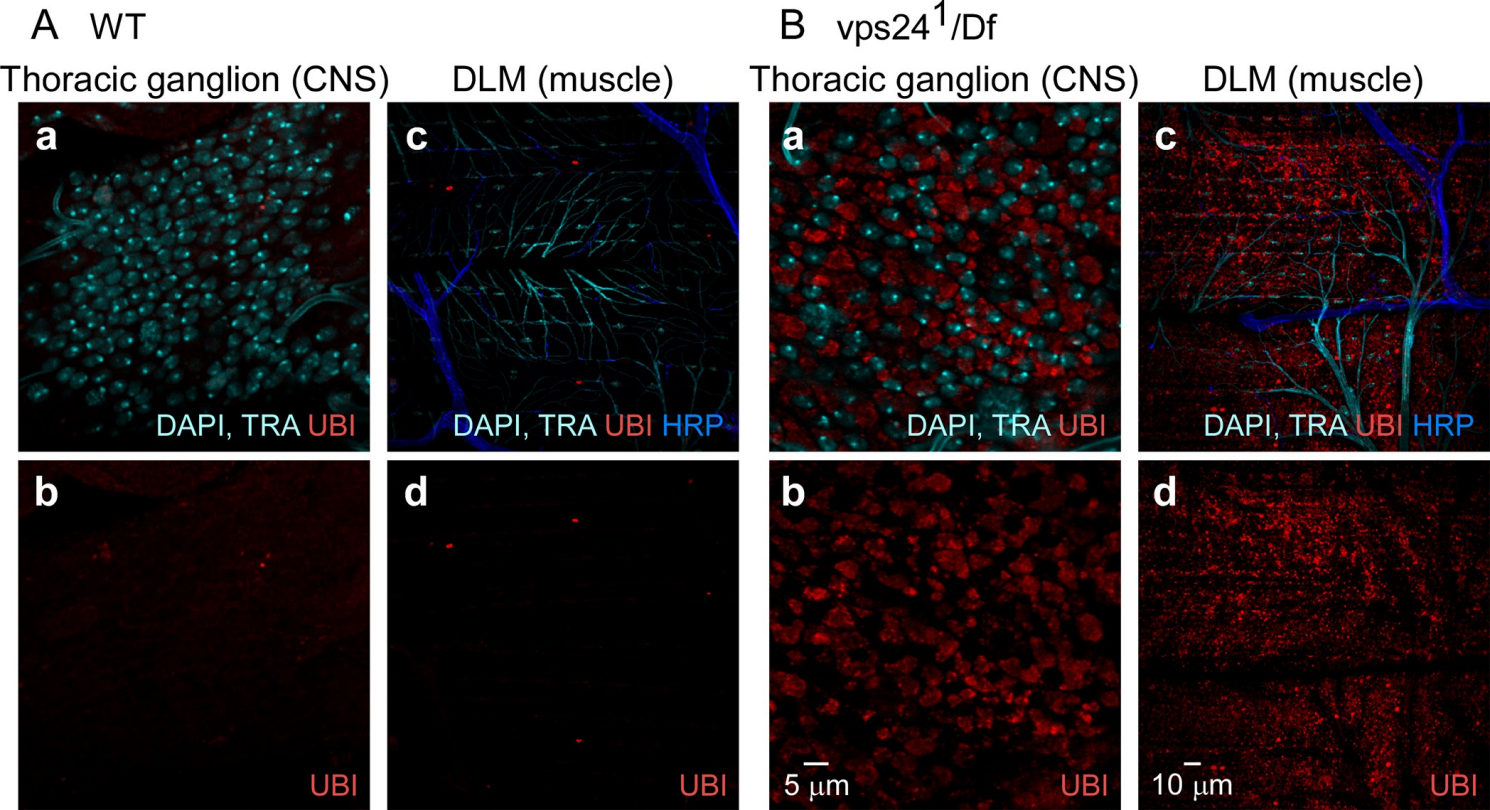
Ubiquitin-positive compartments accumulate in neurons and muscle of the *vps24* mutant. Confocal immunofluorescence images from wild-type (WT) **(A)** or *vps24*^*1*^*/Df(3R)Exel6140* (vps24^1^/Df) mutant flies **(B)**. Ubiquitin (UBI) staining indicates that the *vps24* mutant exhibits a marked increase in ubiquitin-positive compartments in CNS **(a, b)** and muscle **(c, d)** with respect to WT. In contrast to neurons and muscle, ubiquitin positive structures are not prominent in glia of the *vps24* mutant (see S4C and S4D Fig). DAPI labeling of nuclei and autofluorescence from trachea (TRA) appear in the same channel. Anti-HRP labels the neuronal plasma membrane.

Given the established roles of ESCRT function in lysosomal degradation of ubiquitinated proteins, immunocytochemical studies were performed to determine whether the *vps24* mutant exhibits altered proteostasis. Initial studies examined the distribution of ubiquitinated proteins in thoracic tissues involved in motor activity, including the thoracic ganglion of the CNS and the three cell types comprising tripartite DLM neuromuscular synapses [39, 40]. Our previous work has examined these flight motor cell types, including the DLM motor neuron, peripheral perisynaptic glia (PPG) and muscle, in a model for environmental stress-induced failure of proteostasis and degeneration [41]. *vps24*^*1*^ exhibited a striking accumulation of ubiquitin-positive structures in both the CNS and DLM flight muscle at 7 days of age (Fig 3). Further examination of the CNS using cell type-specific markers for neurons and glia (S4 Fig) demonstrated that ubiquitin-positive structures were restricted to neurons and lacking in glia. Notably, a subset of neurons also lacked these structures, including the DLM motor neurons (S4A and S4B Fig), indicating some neuronal cell types are more susceptible to loss of VPS24 function. This was also true among muscle cell types, as indicated by a lack of ubiquitin-positive structures in the nearby coxal muscles of the leg (S5 Fig).

**Fig 4:**
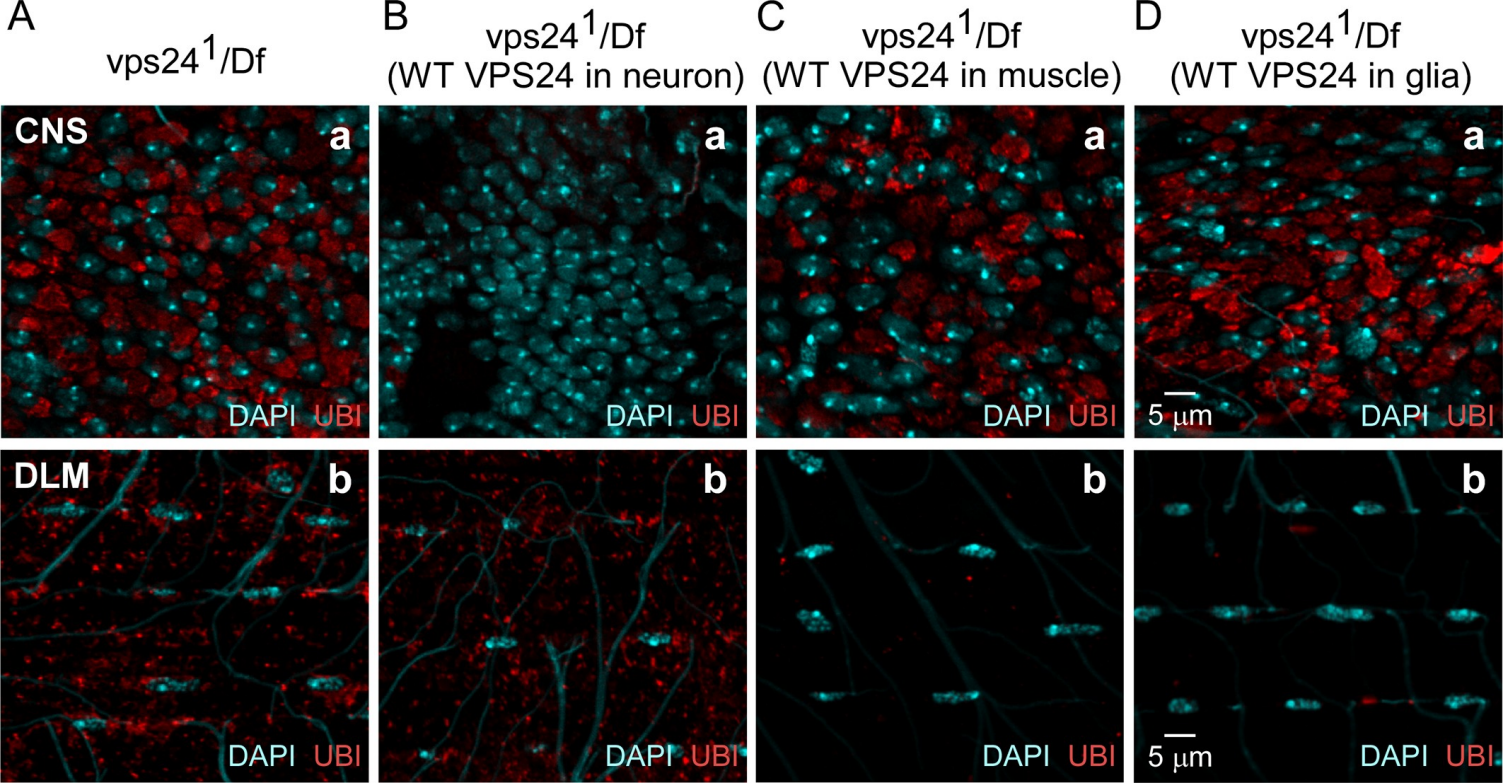
Cell-autonomous rescue and -nonautonomous suppression of ubiquitin-positive compartment accumulation in the *vps24* mutant. Confocal immunofluorescence images of CNS neurons **(a)** and DLM **(b)** of the *vps24* mutant **(A)**, or the mutant rescued by neuronal **(B)**, muscle **(C)** or glial **(D)** expression of the wild-type EGFP-VPS24 protein. Neuronal or muscle expression of wild-type EGFP-VPS24 produces cell-autonomous rescue of the *vps24* mutant phenotype in the respective cell type. Notably, glial expression of wild-type VPS24 produces cell-nonautonomous suppression of the *vps24* phenotype in muscle but not neurons.

To confirm that the preceding cellular phenotypes result from loss of VPS24 function, the CNS and DLM were examined in rescue experiments analogous to those performed in the behavior and lifespan studies. Neuronal expression of wild-type VPS24 produced clear rescue of the neuronal phenotype (Fig 4B), consistent with the observation that ubiquitin-positive structures were restricted to neurons. Similarly, the DLM phenotype was rescued by muscle expression of the same transgene (Fig 4C). Finally, expression of wild-type VPS24 in glia produced a surprising and interesting result (Fig 4D). Whereas the neuronal phenotype was not affected, clear rescue was observed in the muscle and this could not be explained by non-specific transgene expression in muscle (S6 Fig). The preceding findings indicate a cell-autonomous requirement for VPS24 in neurons and muscle, as well as a cell-nonautonomous role for VPS24 in glia.

**Fig 5:**
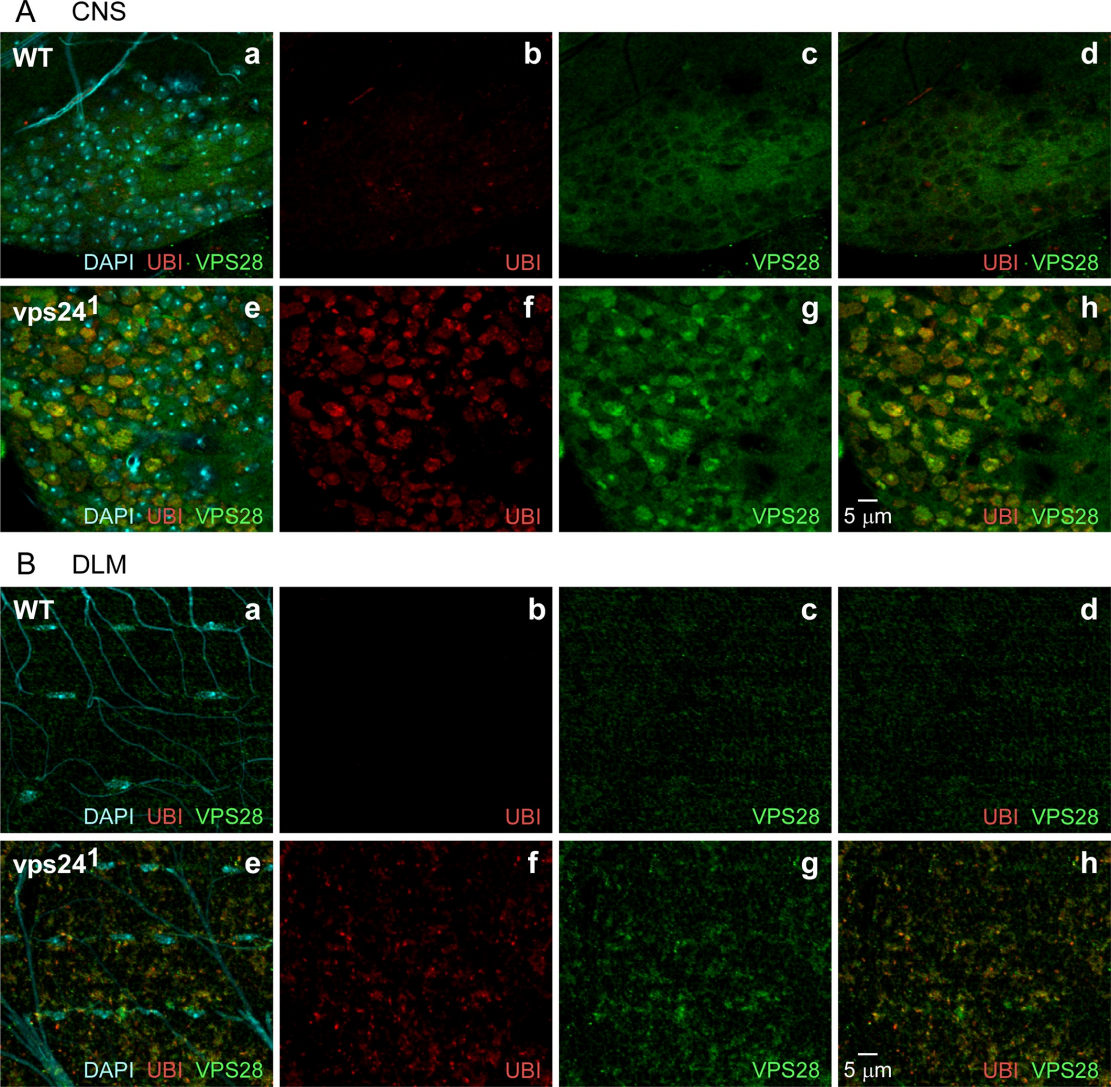
An ESCRT-I component, VPS28, associates with ubiquitin-positive compartments. Confocal immunofluorescence images of CNS neurons **(A)** and DLMs **(B)** from WT **(a-d)** or the *vps24* mutant **(e-h)**. Endogenous VPS28 was detected using an anti-VPS28 antibody. In both neurons and muscle, VPS28 is highly colocalized with ubiquitin-positive compartments and also exhibits a broader diffuse pattern. Only the diffuse VPS28 pattern was observed in neurons lacking ubiquitinated protein deposits. Thus ESCRT-I and III complexes may cooperate in clearance of ubiquitin-positive compartments.

Given the known function of VPS24 as a component of the ESCRT pathway, it was of interest to determine whether the vps24 mutant phenotype includes an altered distribution of ESCRT proteins. This was achieved through immunocytochemical studies of the endogenous VPS28 protein in the *vps24* mutant. VPS28 is a component of the ESCRT-I complex that can interact with ESCRT-III components [42]. In wild-type, VPS28 exhibited a diffuse distribution in both the CNS (Fig 5A, a-d) and DLM (Fig 5B, a-d). In contrast, the VPS28 distribution is altered in the *vps24* mutant and strongly associated with the accumulated ubiquitin-positive structures in neurons (Fig 5A, e-h) and muscle (Fig 5B, e-h). These observations suggest that the cellular *vps24* mutant phenotype involves disruption of ESCRT pathway function leading to aberrant homeostasis in neurons and muscle.

### A role for VPS24 in lysosome homeostasis

**Fig 6:**
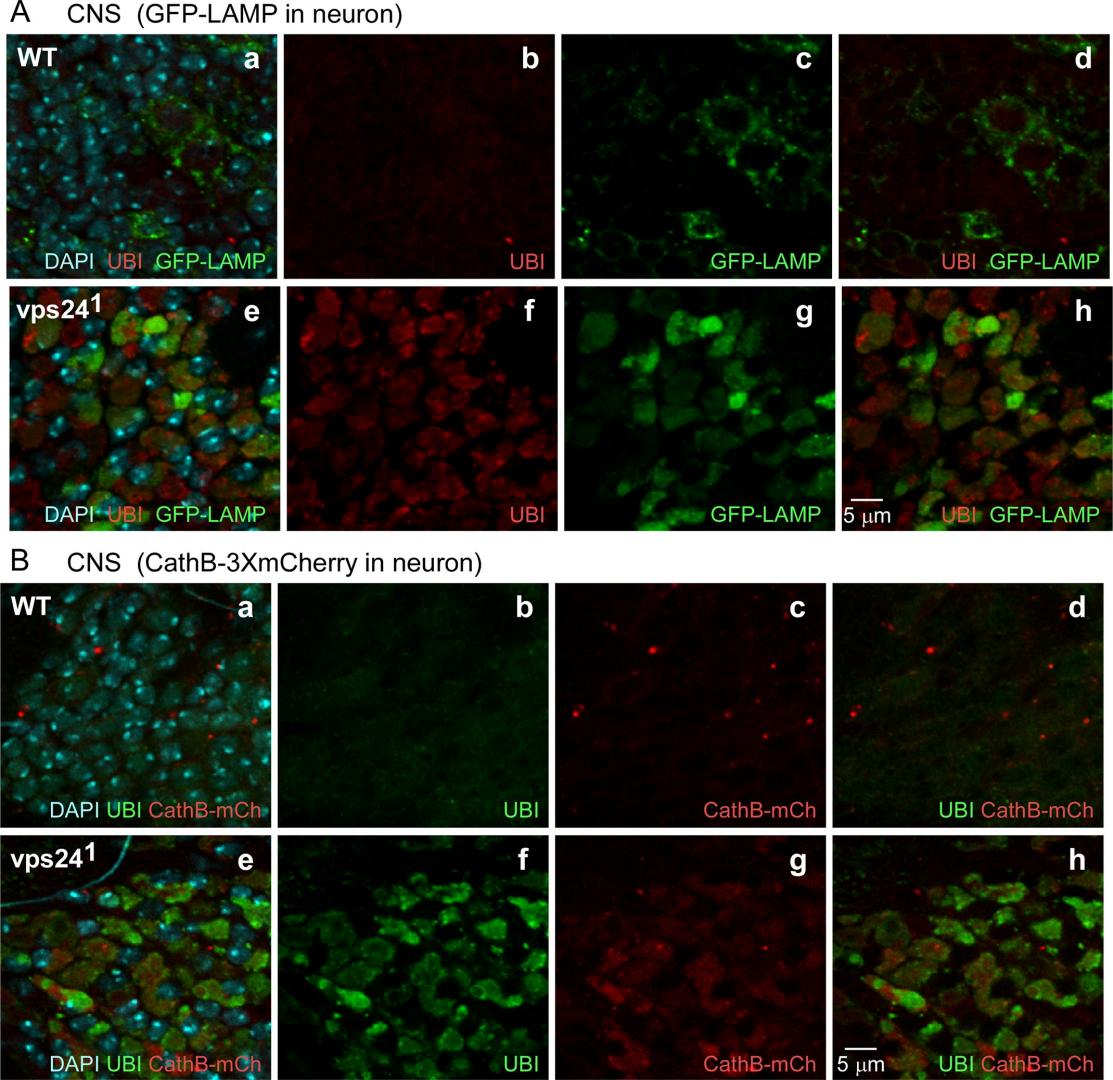
Expansion of lysosomal compartments in neurons of the *vps24* mutant. Confocal immunofluorescence and native GFP or mCherry fluorescence images of CNS neurons from WT **(a-d)** or *vps24* mutant **(e-h)** flies exhibiting neuronal expression of the lysosomal markers, GFP-LAMP **(A)** or Cathepsin-3xmCherry (CathB-mCh) **(B)**. Neurons of the *vps24* mutant exhibited markedly enlarged ubiquitin-positive lysosomal compartments.

To further characterize the ubiquitin-positive structures observed in *vps24* mutant neurons and muscle, their distribution was examined relative to markers for intracellular membrane compartments. These studies revealed clear colocation of accumulated ubiquitin-positive structures with two different lysosomal markers, the lysosome-associated membrane protein 1 (LAMP1) [43–45] and the lysosomal enzyme, Cathepsin B [46, 47]. As shown in Fig 6, neuronal GFP-tagged LAMP1 (Fig 6A) or mCherry-tagged Cathepsin B (Fig 6B) were colocalized with ubiquitin-positive structures in the CNS (note that not all neurons express the transgene). This colocalization was also observed in corresponding fluorescence intensity profiles (S7A and S7B Fig). Additional studies examining Rab7, which is known to associate with late endosomes, autophagosomes and lysosomes [48–53], showed that a Rab7-positive membrane compartment exhibited partial overlap with the ubiquitin-positive lysosomal compartment in *vps24* mutant neurons (S7C and S8A Figs), suggesting accumulation of a late endosomal or autophagosomal compartment in addition to the lysosome. Similar results were obtained when the preceding markers were expressed in the DLM of the *vps24* mutant (S7, S8B and S9 Figs). These findings indicate that the *vps24* mutant exhibits an expanded lysosomal compartment that is present in certain neuronal and muscle cell types and highly enriched in ubiquitinated proteins.

**Fig 7:**
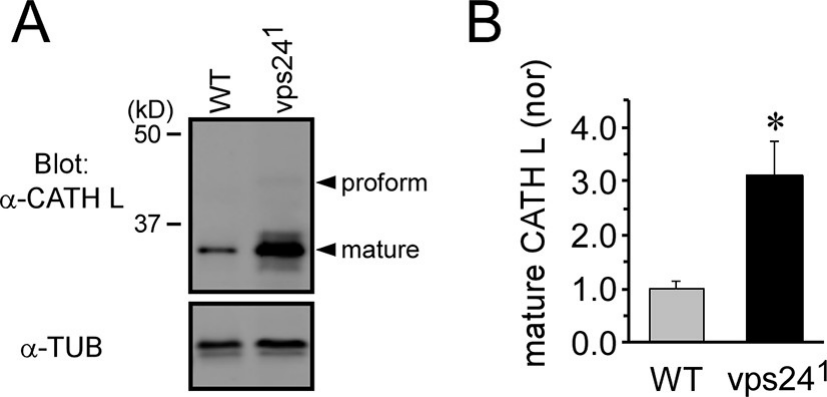
Normal proteolytic processing of Cathepsin L in the *vps24* mutant. **(A)** Western blot analysis of endogenous Cathepsin L (CATH L) showing the proform and processed form of CATH L. Whole fly lysate from WT or the *vps24* mutant was analyzed. In WT, the proform of CATH L is not visible in the image but is present at a detectable level. **(B)** Quantification of processed CATH L. The *vps24* mutant exhibited significant accumulation of processed CATH L (3.1 ± 0.64 fold increase, n = 4) with respect to WT. However, the ratio of proform to processed form was similar in WT and the mutant [WT: 0.023 ± 0.004 (n = 5), 24^1^/Df: 0.013 ± 0.002 (n = 5), *P* = 0.062]. Tubulin (TUB) was used as a loading control.

Further examination of the lysosome compartment in the *vps24* mutant was carried out through Western analysis of the endogenous Cathepsin L protein. As reported previously [54–56], proteolytic processing of the Cathepsin L preprotein occurs in the lysosome where it is dependent upon the acidic luminal pH. Westerns of adult fly extracts from wild-type flies (Fig 7) showed that Cathepsin L was predominantly in the processed form as reported previously (Fig 7A). Notably, the *vps24* mutant exhibited a marked increase in the level of Cathepsin L (Fig 7B), consistent with expansion of the lysosome in this mutant. Moreover, the processed form of Cathepsin L was predominant in the mutant and the ratio of proform to processed form was similar in WT and the mutant (see caption to Fig 7). Although we do not know the status of lysosomal function in this mutant, these results suggest that the lysosome compartment maintains its acidic luminal pH and thus the capacity for proteolytic processing of lysosomal enzymes [54–56].

### Loss of VPS24 function disrupts autophagy

**Fig 8:**
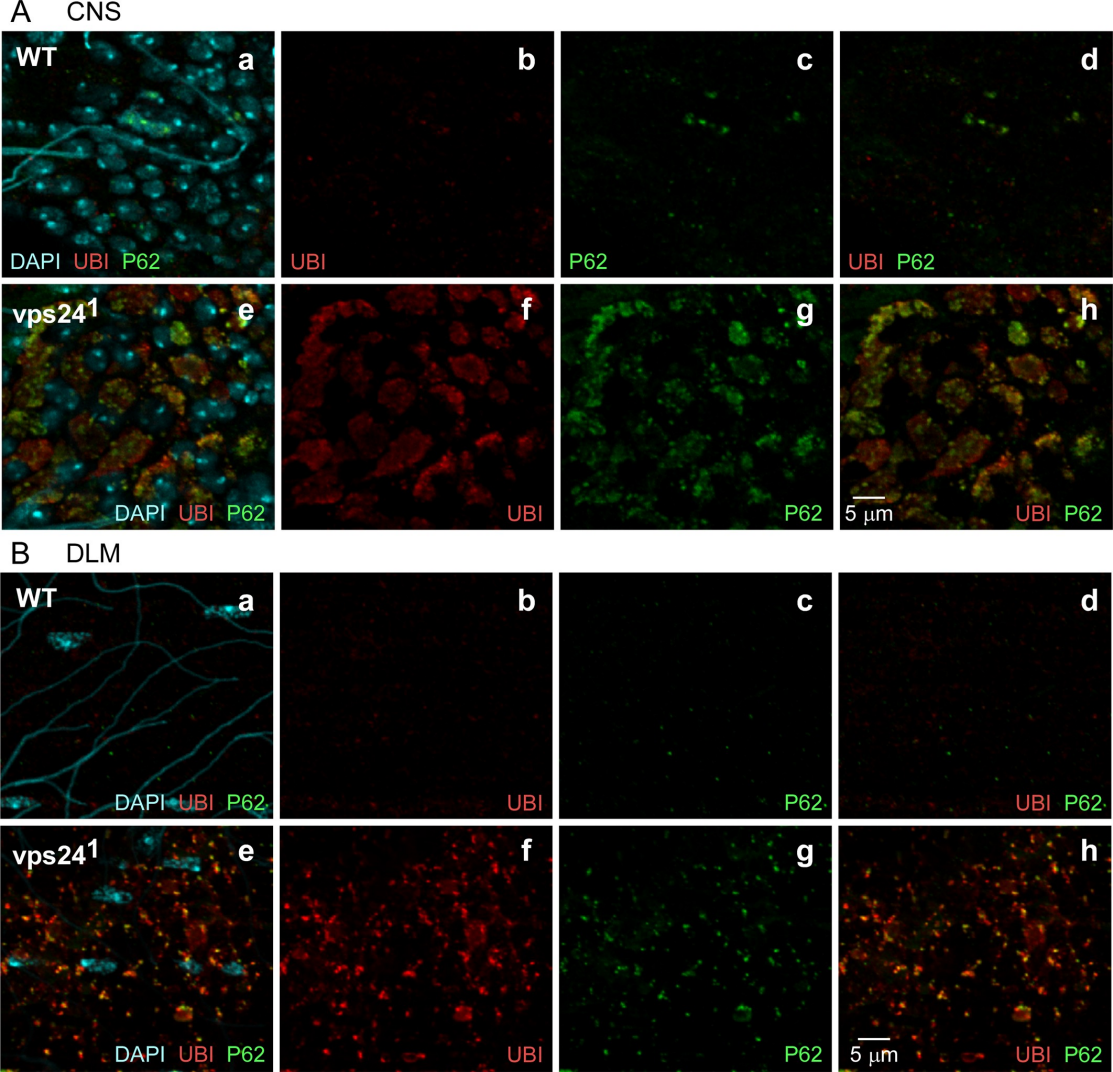
Disruption of autophagy contributes to accumulation of ubiquitin-positive compartments in the *vps24* mutant. Confocal immunofluorescence images of CNS neurons **(A)** and DLM **(B)** from WT **(a-d)** or the *vps24* mutant **(e-h)**. Localization of endogenous P62, the autophagy adaptor protein, was used to mark intermediates in autophagy. In both neurons and muscle, the *vps24* mutant exhibits accumulation of P62. The distribution of P62 overlaps with but is more punctate and restricted than that of the ubiquitin-positive compartment.

VPS24 and the ESCRT pathway are known to participate in autophagic degradation mechanisms [28, 57], that may include lysophagy [58, 59]. To investigate whether autophagy is disrupted in the *vps24* mutant, immunocytochemistry was carried out using markers for autophagic intermediates. The P62 protein plays a key role in autophagy as an adaptor between ubiquitinated proteins and the ATG8a protein associated with nascent autophagophores [60–62]. P62 accumulation is typically interpreted as a disruption of autophagy and accumulation of autophagic intermediates [63, 64]. Examination of CNS neurons (Fig 8A) and DLMs (Fig 8B) of the *vps24* mutant revealed marked accumulation of endogenous P62 and its colocalization with the ubiquitinated lysosomal compartment.

**Fig 9:**
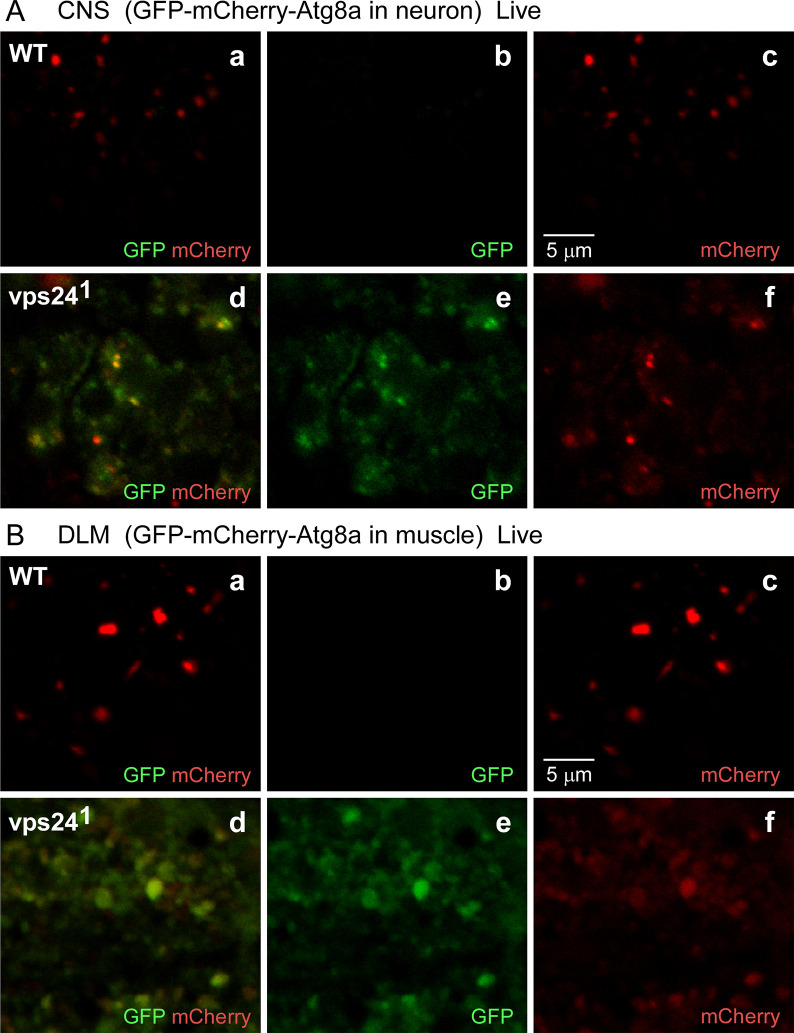
The GFP-mCherry-Atg8a fusion proteins accumulate in a non-acidic compartment in the *vps24* mutant. Confocal live-imaging of the GFP-mCherry-Atg8a fusion protein in CNS neurons **(A)** and DLM **(B)** from WT **(a-c)** or the *vps24* mutant **(d-f)**. Neuronal **(A)** or muscle **(B)** expression of the tandem-tagged fusion protein was achieved as described in Fig 2. In both neurons and muscle of the *vps24* mutant, colocalized GFP and mCherry fluorescence was detected **(Ad-f, Bd-f)**. This indicates that the pH-sensitive GFP tag is not quenched and thus the tandem-tagged fusion protein is in a non-acidic compartment. In WT neurons and muscle **(Aa-c, Ba-c)**, the GFP fluorescence signal is diminished with respect to the mCherry fluorescence.

The functional status of autophagic intermediates in the *vps24* mutant was further examined by live imaging using a genetically-encoded reporter based on another key autophagy protein, ATG8a, which resides in autophagosomes [65, 66]. A variant of ATG8a tagged with a tandem arrangement of GFP and mCherry, GFP-mCherry-ATG8a, reports autophagic trafficking on the basis that GFP, but not mCherry, fluorescence is diminished in an acidic environment [67]. This results from quenching of GFP fluorescence as well as proteolytic lability of GFP in the acidic lumen of the lysosome. In live imaging studies, both red and green fluorescence are observed when the reporter is at neutral pH, such as in the cytoplasm or autophagosome lumen. After autophagosome-lysosome fusion, red fluorescence dominates at the acidic luminal pH of the lysosome. Live imaging studies of the GFP-mCherry-ATG8a reporter were carried out in the CNS and DLM of wild-type and the *vps24* mutant (Fig 9). In wild type, punctate red fluorescence was observed (Fig 9A, a-c and 9B, a-c), suggesting efficient delivery of the reporter to lysosomes. In contrast, a marked increase in green fluorescence was seen in the *vps24* mutant (Fig 9A, d-f and 9B d-f). These findings suggest that autophagic lysosomal degradation is disrupted in the *vps24* mutant. Since the expanded lysosome compartment appears to be acidic (Fig 7), the accumulation of unquenched GFP fluorescence suggests GFP-mCherry-ATG8a resides in a prelysosomal compartment such as the forming or completed autophagosomes.

### Ultrastructural studies of the *vps24* mutant

**Fig 10:**
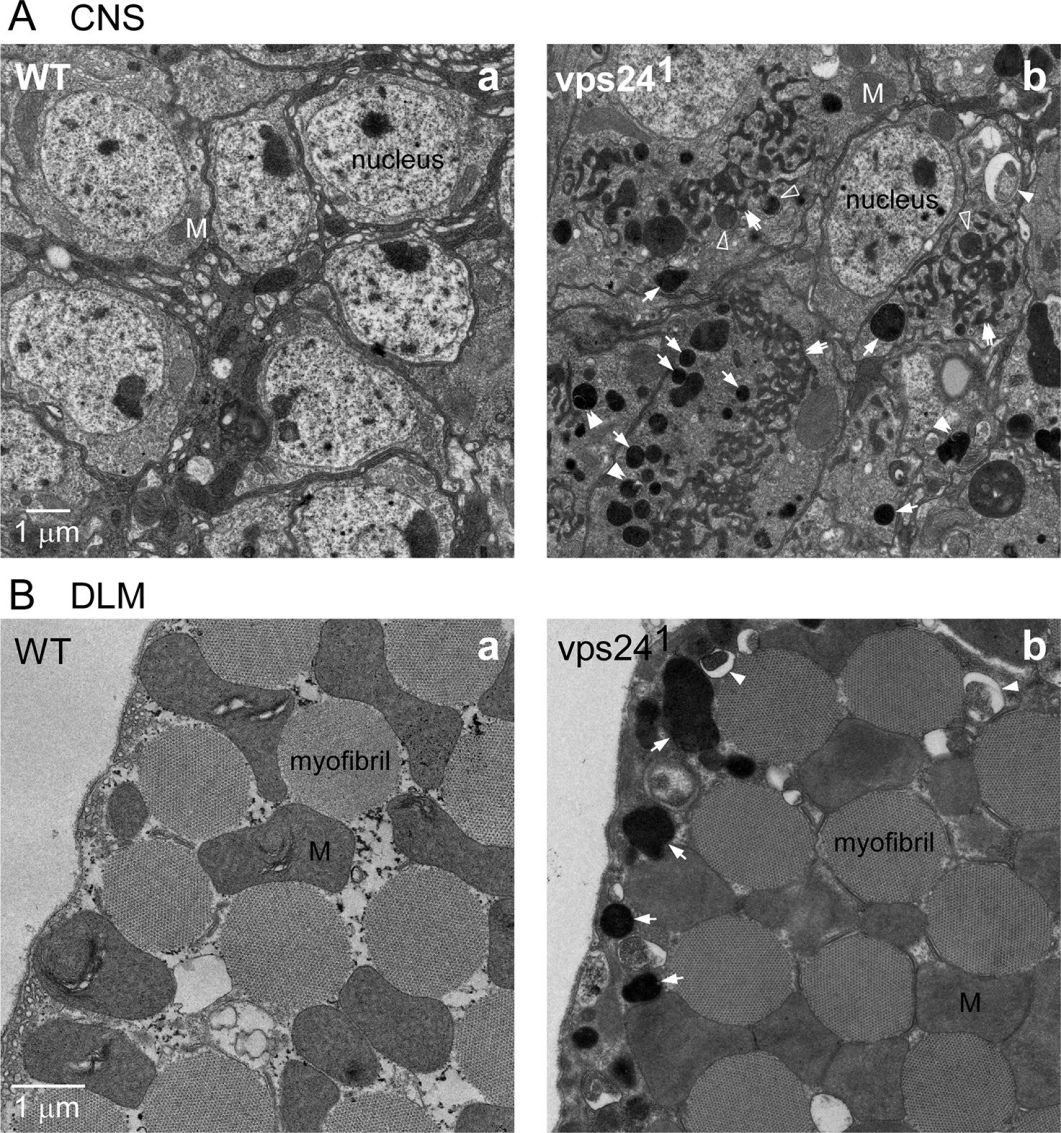
Ultrastructural analysis of the *vps24* mutant. Transmission electron microscopy images of CNS neurons **(A)** and DLM **(B)** from WT **(a)** or the *vps24* mutant **(b)**. The *vps24* mutant exhibited accumulation of autolysosomes (arrows) and tubular membrane compartments (double arrows) as well as autophagosome structures (filled arrowheads), whereas these were absent in WT. The electron dense tubular membrane compartment appeared to be continuous with some electron dense spherical autolysosomes (open arrowheads). In mutant muscle, mitochondria appear to be swollen. Double arrowheads, autophagic intermediates closely associated with spherical autolysosome structures (see S10 Fig); M, mitochondrion.

The preceding findings predict that ultrastructural analysis of the *vps24* mutant phenotype in CNS neurons and DLM flight muscle should reveal an expanded lysosome compartment as well as accumulation of autophagic intermediates. These cell types were examined by Transmission Electron Microscopy (TEM) and found to exhibit striking ultrastructural phenotypes suggesting disruption of lysosome homeostasis and autophagy (Fig 10). Neuronal cell bodies, which are surrounded by a thin layer of cytoplasm in wild type, were dramatically enlarged in the *vps24* mutant and filled with an expanded, electron-dense membrane compartment (Fig 10A). Features of this compartment included a highly electron-dense population of spherical structures (Fig 10Ab) identifiable as autolysosomes on the basis of previous studies [64, 68, 69], as well as a striking tubular network (Fig 10Ab) that appeared to be continuous with some of the spherical autolysosomes (Fig 10Ab). The distribution of autolysosomes and this tubular network throughout the enlarged cell body is consistent with immunocytochemical studies (Fig 6) showing the cell body largely occupied by a ubiquitinated lysosome compartment. As addressed further in the Discussion, the spherical autolysosome-like structures connected to the tubular network were less electron dense than those that appeared to be separate from it, raising the possibility that this tubular network is related to tubular intermediates in lysosome biogenesis or reformation [70–72]. In muscle (Fig 10B), accumulation of autolysosomes occurred near the plasma membrane in the *vps24* mutant (Fig 10Bb), however no pronounced tubular network was observed. These EM studies also revealed accumulation of autophagic intermediates in both CNS neurons and DLM flight muscle of the *vps24* mutant, but not in wild type, consistent with immunocytochemical (Fig 8 and S8 Fig) and live imaging studies (Fig 9). As described previously [64, 68], phagophore or autophagosome intermediates exhibit a characteristic cleft between membrane sheets (Fig 10Ab and 10Bb for neurons and muscle, respectively). Some autophagic intermediates were associated with spherical autolysosome structures (Fig 10Ab, S10 Fig), raising the possibility that lysophagy was disrupted in the *vps24* mutant. Finally, the *vps24* mutant did not exhibit ultrastructural features characteristic of accumulated late endosomes/MVBs [73, 74], consistent with previous studies suggesting that VPS24 may be dispensable for MVB formation [14, 27, 28]. These ultrastructural studies confirm that loss of VPS24 function results in expansion of the lysosomal compartment and disruption of autophagy.

**Fig 11:**
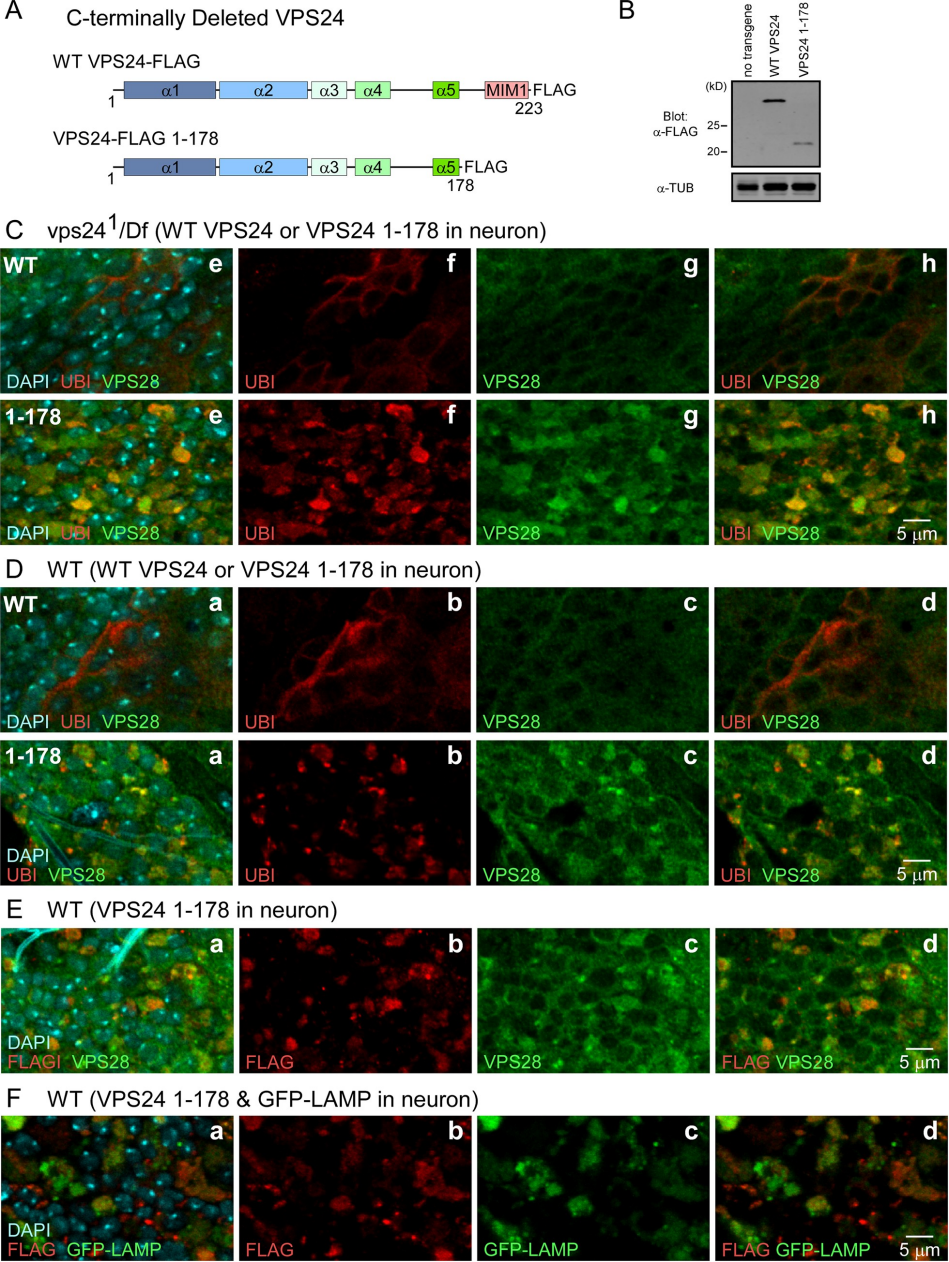
Transgenic expression of a VPS24 mutant lacking the C-terminus disrupts VPS24 function and reveals colocalization of VPS24 with VPS28 at the expanded, ubiquitin-positive lysosomal compartment. (A) Schematic representations of control (WT VPS24-FLAG) and C-terminal truncation mutant (VPS24-FLAG 1–178) VSP24 proteins carrying a C-terminal FLAG tag. The MIM1 [Microtubule-interacting and transport (MIT)-interacting motif 1] domain present in the VPS24 C-terminal mediates binding interactions with MIT domain proteins such as VPS4. The *vps24* mutant form lacks this interaction domain. (B) Western blot analysis of transgene products following neuronal expression of the control or mutant transgene. Lysate was prepared from fly heads and Tubulin (TUB) was used as a loading control. (C-F) Confocal immunofluorescence images of CNS neurons following neuronal transgene expression in a WT or *vps24* mutant background. (C) Neuronal expression of WT VPS24, but not VPS24 1–178, rescues the neuronal phenotype of the *vps24* mutant. (D) Neuronal expression of VPS24 1–178, but not WT VPS24, in a WT background mimics the neuronal phenotype of the *vps24* mutant. (E) Colocalization of VPS24 1–178 and endogenous VPS28 following neuronal transgene expression in a WT background. (F) Colocalization of VPS24 1–178 and the expanded lysosome membrane compartment following neuronal transgene expression in a WT background.

**Transgenic expression a VPS24 mutant lacking the C-terminus mimics the *vps24***^***1***^ **phenotype and reveals colocalization of VPS24 with the expanded, ubiquitin-positive lysosomal compartment.** Studies of human VPS24 [75, 76] have shown that binding of VPS4 to the C-terminal MIM domain is required for ESCRTIII function in membrane remodeling. Overexpression of a VPS24 mutant lacking the C-terminus led to membrane association of the mutant VPS24 and disruption of ESCRT pathway function. In the present study, the *in vivo* functions and interactions of VPS24 were further examined by transgenic expression of the corresponding *Drosophila* VPS24 mutant (VPS24 1–178) that lacks the MIM domain and carries a C-terminal FLAG tag (VPS24 1–178, Fig 11A and 11B). This approach has provided a useful tool for cell type-specific disruption of VPS24 and ESCRT function in a wild-type background. Neuronal expression of this transgene was carried out in a wild-type or *vps24*^*1*^ mutant background, along with a full-length, FLAG-tagged control. The cellular phenotype of the *vps24*^*1*^ mutant was rescued by the control transgene but not VPS24 1–178 (Fig 11C). Notably, expression of VPS24 1–178 in a wild-type background mimicked the *vps24* mutant phenotype by producing accumulation of a ubiquitin-positive compartment in the neuronal cell body (Fig 11D). Moreover, as in the *vps24* mutant, this compartment was associated with endogenous VPS28 (Fig 11D). Under these conditions, the distribution of FLAG-tagged VPS24 1–178 could be examined to determine its spatial relationship to VPS28 (Fig 11E) and the lysosome compartment (Fig 11F). Taken together, these results show that VPS24 and VPS28 are colocalized with the expanded, ubiquitin-positive lysosomal compartment. While we cannot rule out contributions of ESCRT-independent VPS-24 function, these results further indicate that the vps24 mutant phenotype reflects VPS24 interactions with other components of the ESCRT pathway.

## Discussion

The present study provides the first characterization of a *vps24* mutant in a metazoan system and reveals novel phenotypic features with respect to previous genetic analysis of ESCRT mutants. Whereas previously studied *Drosophila* ESCRT mutants have been lethal [10, 13–15, 77–79], this *vps24* mutant is viable and permits post-developmental studies of cell, tissue and organismal homeostasis in the absence of VPS24 function. These have revealed a novel role for VPS24 and the ESCRT pathway in lysosome homeostasis on the basis of the expanded lysosome and associated tubular membrane compartments in the *vps24* mutant. Finally, cell type-specific rescue studies revealed a cell-nonautonomous function for VPS24 in glia, where expression of WT VPS24 can suppress the vps24 loss-of-function phenotype in muscle.

Studies in metazoans have used genetic screens and RNAi-based knockdown (KD) to investigate ESCRT pathway function in several model systems. A previous genetic analysis in *Drosophila* identified and characterized mutants of ESCRTI, ESCRTII and ESCRTIII components and defined their roles using the eye development model [14]. Screening genetic mosaics for eye phenotypes was critical since all the ESCRT mutants recovered were homozygous lethal. ESCRT III components were distinct in that loss of function produced a weaker eye morphology phenotype and, consistent with studies in human cell culture [27, 28], they were dispensable in part for production of MVBs [14]. Notably, this study could not examine mutants of the ESCRTIII component, VPS24, for technical reasons. Thus, the results reported here provide additional insight into the *in vivo* functions of VPS24 and the ESCRT pathway in metazoans.

### Cellular and molecular mechanisms of VPS24 function

At the cellular level, expansion of the lysosome compartment in neurons and flight muscle was the most striking *vps24* mutant phenotype. While this is a novel phenotype for an ESCRT mutant, it may also intersect with previous studies of ESCRT proteins in yeast [80, 81]. In neurons of the *Drosophila vps24* mutant, accumulation of both spherical autolysosomes and an associated membrane compartment with a tubular network morphology raises new possibilities regarding a potential role for VPS24 and ESCRTs in lysosome homeostasis. In considering the underlying mechanisms, the *vps24* mutant phenotype exhibiting both disruption of autophagy and accumulation of autolysosomes might indicate that autolysosomes are nonfunctional and cannot process their contents, even under conditions of reduced autophagic flux [68, 82, 83]. Alternatively, autolysosome accumulation may result from compromised autophagic mechanisms of lysosome turnover through lysophagy [58, 59]. In the *vps24* mutant, disruption of lysophagy might result in expansion of a ubiquitinated lysosomal compartment marked with P62 (Fig 8). Finally, autolysosome accumulation might result from disruption of lysosome reformation. We will discuss this third potential mechanism further because it may explain marked accumulation of the tubular membrane compartment in neuronal cell bodies.

A striking feature of the *vps24* mutant phenotype is observed at the ultrastructural level as marked accumulation of a tubular membrane compartment in neuronal cell bodies as well as spherical autolysosomes (Fig 10Ab). On the basis of apparent connections between autolysosomes and the tubular network, and the electron dense appearance of both compartments, the latter may represent tubular intermediates in lysosome biogenesis. Characterization of tubular lysosomal structures in previous studies [71] has shown that autolysosomes produce new lysosomes through tubular intermediates [70, 72]. Notably, disruption of this process leads to accumulation of autolysosomes and tubular intermediates [84] as described here for the *vps24* mutant. One possibility is that the process is arrested in the *vps24* mutant before tubular intermediates are resolved to form new lysosomes. This would explain why some spherical autolysosomes appear to connect with the tubular network and are less electron dense than those that are separated (Fig 10Ab). Such a mechanism would suggest a novel role for VPS24 and ESCRT function in a membrane fission process that converts tubular intermediates to new lysosomes, possibly in a Clathrin- and Dynamin-dependent fashion [70, 72, 84, 85]. In this regard, localization of the ESCRT proteins, VPS24 and VPS28, to the accumulated ubiquitin-positive lysosome (Figs 5 and 11) would be consistent with a direct function in membrane remodeling of this compartment. While a role for VPS24 in this process would not represent a canonical ESCRT mechanism involving membrane compartment formation away from the cytoplasm, it would be compatible with recent studies of an analogous ESCRTIII-dependent membrane remodeling reaction in peroxisome biosynthesis [18] and endosome recycling [17].

The preceding mechanisms would not be mutually exclusive, rather a combination may be important in maintaining lysosome morphology and function. For example, failure of VPS24 to resolve tubular lysosome intermediates and aberrant accumulation of the tubular network may lead to its ubiquitination and targeting for lysophagy. Consequently, an ineffective autophagy pathway may exacerbate lysosome expansion, contributing to marked accumulation of a lysosome compartment marked with ubiquitin and P62.

### An organismal perspective on VPS24 function

Features of the cellular phenotypes observed in the *vps24* mutant have important implications at the organismal level. First, the cell type-specificity of the phenotypes observed in certain neuronal and muscle cells (Fig 3, S4 and S5 Figs) indicates striking differences among cell types in their requirements for VPS24 function. Second, the ability of VPS24 to act cell non-autonomously in glia to suppress the *vps24* mutant phenotype in flight muscle, but not neurons (Fig 4D), reveals a function for VPS24 in systemic intercellular signaling.

The *vps24* mutant phenotypes are largely restricted to certain cell types. These include a striking lysosome expansion phenotype that is present only in neuronal cell types and absent from glia. In addition, lysosome dysfunction was observed in the flight muscle but not the coxal muscle of the leg. Notably, VPS24 function in neurons is critical for motor function and viability of the organism (Fig 2), indicating that susceptibility of neurons to loss of VPS24 function has important consequences. Such cell type-susceptibility is a common feature of neurodegeneration and has been an important focus in studies of neurodegenerative diseases [86, 87]. These include a wide range of lysosomal storage disorders [88, 89] and diseases caused by ESCRTIII dysfunction [36, 37].

Finally, we report a cell-nonautonomous function for VPS24 that originates in glia and compensates for loss of VPS24 function in muscle but not neurons (Fig 4D). In contrast, VPS24 function in neurons or muscle does not exhibit such a cell non-autonomous function (Fig 4B and 4C). Conversely, glia do not show a cell autonomous requirement for VPS24 in lysosome homeostasis (S4 Fig). The ESCRT pathway has been implicated in intercellular signaling mechanisms in a wide range of experimental systems [7]. This is often attributed to ESCRT-mediated production of extracellular vesicles in the form of exosomes derived from multivesicular bodies or ectosomes. Recent studies in *Drosophila* have demonstrated such an ESCRT-mediated mechanism in glia, where exosomes deliver micro-RNAs to other cell types to regulate neuromuscular synapse growth and tracheal branching [90]. In the flight motor, our previous work revealed a form of cell non-autonomous signaling from muscle, in which muscle-specific expression of a small heat shock protein protects neurons and glia against degeneration induced by environmental stress [41]. Future studies in the flight motor will address whether common mechanisms may operate in these distinct cell-nonautonomous signaling functions and further investigate the role of the ESCRT pathway.

The novel functions reported here for VPS24 and ESCRTs in lysosome homeostasis and intercellular signaling may have important implications for our understanding of ESCRT-dependent mechanisms as well as potential therapeutic approaches for neurodegenerative disease. Recent models proposing tubular intermediates in lysosome biogenesis have not included a role for ESCRT function [70, 72]. The present study introduces an additional mechanism that may contribute to this process and future work is expected to further define roles for the ESCRT pathway in lysosome homeostasis. Moreover, understanding the molecular basis of cell non-autonomous VPS24 function in glia, including the extracellular signaling molecules and response mechanisms in target cells, may have implications for treatment of neurodegenerative diseases related to aberrant ESCRT or lysosomal function.

## Materials and methods

### *Drosophila* strains

Wild-type *Canton-S* flies, as well as *Appl-GAL4* and *w;;Ly/TM6c* were from our laboratory stock collection. *Mhc-Gal4*, *Repo*-Gal4, *UAS-GFP*, *UAS-GFP-mCherry-atg8a*, *UAS-GFP-Rab7*, *Df(3L)GN34*, *Df(3R)Exel6140* and a P element allele of *vps24*, *yw;;P w*^*+*^*y*^*+*^ *vps24[EY04708]*, were obtained from the Bloomington Stock Center (Indiana University, Bloomington, IN). *UAS-EGFP-vps24*, *UAS-vps24-FLAG* and *UAS-vps24 1–178 FLAG* transgenic lines were generated in this study (see “Generation of transgenic lines”). *UAS-GFP-LAMP* [43], *UAS-CathB-3XmCherry* [46] and *D42-Gal4 Cha-Gal80* [91] transgenic lines were kindly provided by Dr. Helmut Krämer (University of Texas Southwestern Medical Center, Dallas, TX), Dr. Gábor Juhász (Eötvös Loránd University, Hungary) and Dr. Fernando Vonhoff (Yale University, New Haven, CT), respectively. Stocks and crosses were cultured on a conventional cornmeal-molasses-yeast medium at 20°C in a 12 hour day-night cycle. All experiments were carried out using virgin female flies.

### Mutagenesis and screening

A genetic screen was carried out carried out essentially as described previously [29]. Briefly, *Canton-S* males carrying an isogenized third chromosome (Iso3) were exposed to 25 mM ethyl methanesulfonate (EMS) for 24 h. F2 flies heterozygous for a mutagenized third chromosome *in trans* to the third chromosome carrying deficiencies, *Df(3L)GN34* and *Df(3R)Exel6140*, were screened for motor defects at 38°C.

### Behavioral analysis

TS paralytic behavior was examined as described previously [92]. Groups of six flies were placed in a vial preheated to 38°C by immersion in a water bath. Five groups were examined for each genotype. Time for 50% paralysis represents the time at which three flies were no longer able to stand. Tests were truncated at 5 min if 50% paralysis had not occurred. Climbing tests were performed as described previously [41]. Groups of six flies were tapped to the bottom of a 100-ml graduated cylinder, and the time for half of the flies to climb to the 40 ml mark (7.3 cm) was recorded. For each test, this process was repeated three times to produce a mean value. The mean values were averaged over multiple tests, and the inverse was taken to obtain a climbing index (CI) such that larger values represent faster climbing. The climbing tests were truncated at 2 min if 50% climbing had not occurred and zero was given for the climbing index. CI values represent data from tests on ten independent groups of six flies. Behavioral studies were carried out on 7d old female flies raised at 20°C.

### Lifespan assay

Lifespan assays were performed essentially as described [93]. Virgin female flies were collected and transferred to fresh vials every other day. For each transfer, deaths were scored until all flies were dead. For each genotype, 50 flies were used to generate the survival curve.

## Molecular characterization of the *vps24*^*1*^ mutant

Sequence analysis of the *vps24* gene region was carried out as described previously [94]. Briefly, genomic DNA was prepared from the *vps24*^*1*^ mutant and used as template for PCR. Gel-purified PCR products were sequenced at the Penn State University Nucleic Acids Facility. Sequences from the *vps24*^*1*^ mutant were compared to those from the parent third chromosome used in the mutagenesis.

To examine transcripts, total RNA was isolated from whole flies using Trizol reagent (Life Technologies, Carlsbad, CA) according to the manufacturer’s protocol and then reverse transcribed using qScript cDNA synthesis kit (Quanta Biosciences, Gaithersburg, MD). The resulting cDNA preparations were used as a template for the PCR assay and sequencing. Gel-purified PCR products were sequenced at the Pennsylvania State University Nucleic Acids Facility. The primer set used in the PCR produces 400bp products with the cDNA of wild-type unspliced transcript as a template. Primers used: vps24-F (at the beginning of the *vps24* ORF residing in Exon 1): 5′ ATGGGCTTATTCGGCAGAAC 3′; vps24-R: 5′ GCATCTGAATGGAGTTGAGG 3′

### Quantitative-PCR (qPCR)

Extraction of total RNA from whole flies using Trizol (Life Technologies) was followed by synthesis of complementary DNAs. Reverse transcription of 600 ng of RNA in a 30 μl reaction volume was carried out using the qScript cDNA synthesis kit (Quanta Biosciences). The resulting cDNA sample was diluted 6-fold. For the qPCR assay, 4 μl of the diluted cDNA sample was used for a 20 μl reaction volume according to the manufacturer’s instructions for SYBR Green PCR Master Mix (Applied Biosystems). Primers were designed based on *D*. *melanogaster* mRNA sequences obtained from FlyBase. They were imported into Primer Express Software (Applied Biosystems) to generate primer pairs suitable for qPCR. Amplification efficiency was tested for each primer pair before quantitation experiments.

The qPCRs were run on the ABI StepOne Real-Time PCR instrument (Applied Biosystems) using the following cycling conditions: The quantitative PCR program was as follows: 10 min at 95°C, 40 cycles (15 s at 95°C, 60 s at 60°C). After the 40 cycles, the dissociation curves of the amplicons were obtained to verify adequate amplification of the target sequence. Transcript levels of each target gene were calculated using the ΔCt method of relative quantification using transcript levels of the ribosomal protein 49 (rp49) for normalization across samples. Four separate samples were prepared from each genotype.

Real-Time PCR Primers. Listed below are the primers that were used for qPCR analysis of the cDNAs for *Drosophila vps24* and *rp49*.

vps24 fwd: TTCTTGCCAAGGAGATTGTGAA

vps24 rev: GGTGCGCCTTCGACGTATAT

rp49 fwd: GCAAGCCCAAGGGTATCGA

rp49 rev: ACCGATGTTGGGCATCAGA

### Generation of transgenic lines

Three new transgenic lines were generated for expression of VPS24 proteins, including a C-terminally truncated version that interferes with VPS24 function in a wild-type background. A transformation construct for the *UAS-EGFP-vps24* transgenic line was generated by fusing the open reading frame (ORF) of *vps24* with the EGFP ORF to create an N-terminal EGFP tag. This product was inserted into the P element transformation vector, pUAST [35]. A cDNA clone for *vps24* was obtained from the *Drosophila* Genomics Resource Center (clone ID: GH14561, corresponding cDNA accession number: AY058381). The *vps24* ORF, including the flanking BglII and KpnI restriction sites, was amplified by PCR using Pfu DNA polymerase. These restriction sites were used to shuttle the *vps24* ORF into pBluescriptI SK- (pBlue) and ligated with a pre-existing EGFP sequence in pBlue. The EGFP-vps24 segment was sequentially digested with NotI and KpnI and shuttled into the P element transformation vector, pUAST. UAS-vps24-FLAG was generated from an existing *vps24* transgene construct by inserting the FLAG coding sequence after the *vps24* ORF. This construct was modified to produce UAS-*vps24 1–178* FLAG by replacing the full length *vps24* ORF with a PCR product corresponding to the truncated coding sequence. Generation of transgenic lines was achieved as described previously [95].

### Synaptic electrophysiology

Excitatory postsynaptic currents (EPSCs) were recorded at dorsal longitudinal flight muscle (DLM) neuromuscular synapses of 3 to 5 day-old adults reared at 20°C. These experiments were performed as described previously [34, 96]. Briefly, two-electrode voltage clamp was performed with a TEV-200 amplifier (Dagan Corporation, Minneapolis, MN). The recording solution consisted of (in mM): 128 NaCl, 2 KCl, 4 MgCl_2_, 1.8 CaCl_2_, 5 HEPES, and 36 sucrose. The pH was adjusted to 7.0 using NaOH. Temperature control was achieved using a TC-202A temperature controller and PDMI-2 microincubator (Harvard Apparatus, Holliston, MA). Data were acquired using Pulse software (Heka Electronik, Lambrecht, Germany) and an ITC-16 laboratory interface (Instrutech, Great Neck, NY). Stimulation of DLM motor axons was achieved with a Master-8 Stimulator (A.M.P.I., Jerusalem, Israel). Synaptic currents were low-pass filtered at 5 kHz and acquired at 25 kHz. Measurements of synaptic current amplitudes was carried out in the Mini Analysis Program (Synaptosoft, Decatur, GA).

### Immunocytochemistry and confocal microscopy

Immunocytochemistry and confocal microscopy were performed essentially as described previously [34]. DLM flight neuromuscular tissue together with the thoracic ganglia were imaged using an Olympus FV1000 confocal microscope (Olympus Optical, Tokyo, Japan) with a PlanApo 60x 1.4 numerical aperture oil objective (Olympus Optical) and a z-step size of 0.2 μm. Typically, maximum projections of two consecutive optical z-sections were shown. The same methods were utilized to examine coxal muscle neuromuscular synapses in the leg. Live imaging of GEP-mCherry-Atg8a was performed with a LUM Plan 60X 1.0-NA water immersion objective (Olympus Optical) as described previously [94, 97]. Images were obtained and processed with Fluoview software (Olympus Optical). Images shown are representative of those obtained from at least 4 different preparations. Experiments were carried out on 7d old female flies raised at 20°C.

Fluorescence intensity profiles were utilized to further examine colocalization in confocal images. Using the Image J software package (NIH), these were generated from a line of pixels running horizontally across the middle of the image. Pixel intensity values were normalized to the maximum pixel intensity following subtraction of the minimum pixel intensity and the normalized values were plotted as a function of distance across the image.

Antibodies and reagents: Primary antibodies included mAb FK2 α-Ubiquitin (1:1000) (Enzo Life Sciences, Farmingdale, NY); mAb M2 α-FLAG (1:10,000) (Sigma-Aldrich); rabbit α-VPS28 (1:2000) [13] [Dr. Helmut Krämer (University of Texas Southwestern Medical Center, Dallas, TX)]; rabbit α-P62 (1:5000) [98] [Dr. Gábor Juhász (Eötvös Loránd University, Hungary)]; goat α-HRP-Alexa647, which labels neuronal plasma membranes (1:200) (Jackson Immunoresearch Laboratories, West Grove, PA). Secondary antibodies included Alexa Fluor 488-conjugated anti-mouse IgG (1:200), Alexa Fluor 568-conjugated anti-mouse IgG (1:200) and Alexa Fluor 568-conjugated anti-rabbit IgG (1:200) (Invitrogen). Nuclear staining was performed using DAPI (300 nM) (Sigma-Alrich, St. Louis, MO).

### Western analysis

Western analysis of fly homogenates was performed using conventional methods, as described previously [99]. For the analysis of Cathepsin L, the equivalent of 0.1 fly bodies was loaded per lane on a 9% SDS-PAGE gel. In case of neuronal expression of VPS24-FLAG transgene products, the equivalent of 0.4 fly heads and 12% SDS-PAGE gels were used. The studies utilized the following Primary antibodies: mAb 193702 α- insect Cathepsin L (MAB22591; 1 μg/ml; R&D Systems, Inc. Minneapolis, MN), mAb M2 α-FLAG (F3165; 1:500; Sigma-Aldrich), mAb 6-11B-1 α-Tubulin (T6793; 1:100,000; Sigma-Aldrich). The secondary antibody was IRDye 680RD Donkey α-Mouse IgG (925–68022; 1:5,000; LI-COR Biosciences, Lincoln, NE). Detection was performed using a LI-COR OdysseyCLx imager (LI-COR Biosciences). Images were analyzed using Image Studio software (LI-COR Biosciences). Western results shown are representative of at least four independent experiments. Experiments were carried out on 7d old female flies raised at 20°C.

### Transmission electron microscopy

TEM studies employed conventional methods, essentially as described previously [33, 100]. Briefly, tissues were dissected in saline solution and fixed in the same saline containing 2.5% paraformaldehyde and 1.5% glutaraldehyde. Samples were postfixed in 1.0% osmium tetroxide for 2 hrs, en bloc stained in 2% aqueous uranyl acetate for 1 hr, dehydrated using an ethanol series, and embedded in Spurr’s resin (EM Sciences, Fort Washington, PA). 75 nm sections were mounted on mesh copper grids followed by uranyl acetate/lead citrate staining before viewing at 120 kV on a FEI Tecnai G2 Spirit BioTwin microscope equipped with FEI Eagle 4k HS CCD camera (FEI Company, Hillsboro, OR). Images shown are representative of those obtained from at least 3 different preparations. Experiments were carried out on 7d old female flies raised at 20°C. EM studies were carried out in the Penn State University Electron Microscopy Facility.

### Analysis of numerical data

Microsoft (Seattle, WA) Excel was utilized to analyze numerical data and generate graphs. All data values are presented as mean ± SEM. Statistical significance was determined using the two-tailed Student’s *t* test and significance was assigned to comparisons for which *P* ≤ 0.05.

## Supporting information

S1 Fig A forward genetic screen for mutations affecting synaptic transmission and recovery of a new mutant of *vps24*. **(A)** Male flies with an isogenized third chromosome (Iso3) were exposed to ethylmethane sulphonate (EMS). Mutagenized males were mated with females carrying the third chromosome marker, Lyra (Ly), in trans to a balancer chromosome, TM6c. F1 males carrying a mutagenized third chromosome (3*) in trans to TM6c were crossed to females with the third chromosome deficiencies, *Df(3L)GN34* and *Df(3R)Exel6140*. F2 progeny carrying 3* in trans to the deficiency chromosome were screened for motor defects at 38°C. **(B)** *748/Df(3R)Exel6140* flies (748/Df) exhibited rapid paralysis at 38°C, whereas wild-type flies (WT) did not. Tests of *748/+* flies indicated the phenotype is recessive. Tests were truncated at 5 min if 50% paralysis had not occurred. Here and in subsequent figures, data points represent the mean ± SEM and asterisks mark significant differences from control values (*P* = 0.05). **(C-E)** Complementation testing. **(C)** The deficiency within the right arm, *Df(3R)Exel6140*, failed to complement 748, whereas *Df(3L)GN34* did not. Further complementation testing was performed using six existing mutants that disrupt genes within *Df(3R)Exel6140* (boxed in red). **(D, E)** A P element insertion in the *vps24* gene, *vps24[EY04708]* (red arrowhead in **D**, vps24 P in **E**), failed to complement, indicating that the *748* mutation resides in *vps24*.
(TIF)

S2 Fig Aberrant *vps24*^*1*^ transcripts. **(A)** PCR using *vps24*^*1*^ cDNA as a template (lane 3) produced 2 kinds of products. The upper band migrated at similar molecular mass to that observed when using the mutant genomic DNA as a template (lane 6). The lower band migrated at a slightly lower molecular mass in comparison to that from wild-type *vps24* cDNA (the major band in lane 2). The faint upper band in lane 2 corresponds to the 400 bp PCR product expected from the unspliced *vps24* transcript in WT. The 2 kinds of PCR products from the *vps24*^*1*^ cDNA were gel-purified and sequenced (see Fig 1D). RT-: cDNA synthesis reaction without reverse transcriptase (RT). **(B)** Quantitation of *vps24* transcript levels by qPCR. The blue bar under the *vps24* transcript diagram indicates the location of amplicon examined. The *vps24* transcript level of WT control (+/Df) was set at 1.0. The P element insertional allele of *vps24* exhibited reduction of the transcript levels by more than 50% relative to WT or *vps24*^*1*^.
(TIF)

S3 Fig The *vps24* mutant exhibits wild-type synaptic transmission at the dorsal longitudinal flight muscles (DLMs) neuromuscular synapses. Two-electrode voltage-clamp recordings of excitatory postsynaptic currents (EPSCs) from DLM neuromuscular synapses of WT and the *vps24* mutant at permissive **(A-C)** and elevated **(D-F)** temperatures. Representative recordings **(A, D)** and peak amplitude measurements of EPSCs **(B, E)** indicate wild-type EPSC waveform and amplitude in the *vps24* mutant. Stimulation artifacts were removed for clarity. **(C, F)** The *vps24* mutant synapses exhibit wild-type short-term depression during train stimulation at 1 or 20 Hz. Peak EPSC amplitudes were normalized to the initial amplitude and plotted as a function of stimulus number.
(TIF)

S4 Fig Ubiquitin-positive structures in the *vps24* mutant are restricted to neurons. Confocal immunofluorescence and native GFP fluorescence images from the *vps24* mutant flies exhibiting neuronal or glial expression of soluble GFP proteins. **(A)** CNS Neurons labeled by expression of GFP using the *Appl-Gal4* neuronal driver. An accumulation of ubiquitin-positive structures was observed in the majority of neurons, though some neurons (arrows) lack these structures. Soluble GFP filled the cytoplasm as well as the nucleus. Note that not all neurons express the transgene. **(B)** A motor neuron innervating DLM fibers was selectively labeled by expression of GFP using the *D42-GAL4 Cha-GAL80* double driver. No ubiquitin-positive structures were observed in the cell body (arrow). **(C)** CNS glia labeled by expression of GFP using the *Repo-Gal4* glial driver. Ubiquitin-positive structures were lacking in CNS glia (arrows). **(D)** Peripheral Perisynaptic Glia (PPG) labeled by expression of GFP using the *Repo-Gal4* glial driver. A cell body of PPG which resides in the periphery over the DLM surface (arrow) lacked ubiquitin-positive structures.
(TIF)

S5 Fig Lack of ubiquitin-positive structures in leg muscle in the vps24 mutant. Confocal immunofluorescence images of leg (coxal) muscle from WT (A) or the vps24 mutant flies (B). The coxal muscles from the vps24 mutant showed no accumulation of ubiquitin-positive structures and resembled WT.
(TIF)

S6 Fig Cell type-specific expression of EGFP-VPS24 transgene. Confocal immunofluorescence and native GFP fluorescence images of DLM from the *vps24* mutant flies exhibiting muscle or glial expression of wild-type EGFP-VPS24 protein. **(A)** Muscle expression of wild-type EGFP-VPS24 produced a clear EGFP-VPS24 signal in the DLM that resembled the muscle contractile apparatus. **(B)** Glial expression of wild-type EGFP-VPS24 produced no detectable EGFP-VPS24 signal in the DLM but resulted in cell-nonautonomous suppression of the DLM *vps24* mutant phenotype.
(TIF)

S7 Fig Fluorescence intensity profiles examining colocalization. Comparisons of pixel intensity profiles for Ubiquitin with the lysosomal markers (GFP-LAMP) **(A)** and Cathepsin B (Cathepsin-3xmCherry, CathB-mCh) **(B)** or the late endosome, autophagosome and lysosome protein, Rab7 (GFP-Rab7) **(C)** in the *vps24* mutant. Images for the CNS correspond to those in Fig 6 and S8A Fig, respectively. Images for the DLM correspond to those in S8B and S9 Figs, respectively. A white line shown in each image designates a line of pixels whose intensities were measured. These values were normalized to the maximum pixel intensity following subtraction of the minimum pixel intensity, and plotted as a function of distance. In both the CNS and DLM, GFP-LAMP and CathB-mCh were colocalized with ubiquitin-positive structures. In contrast, GFP-Rab7 overlaps partially with the ubiquitin-positive lysosomal compartment.
(TIF)

S8 Fig The Rab7 marker exhibits partial overlap with ubiquitin-positive lysosomal structures in the *vps24* mutant. Confocal immunofluorescence images of CNS neurons **(A)** and DLMs **(B)** from WT **(a-d)** or *vps24* mutant **(e-h)** flies exhibiting expression of GFP-Rab7 in the corresponding tissues.
(TIF)

S9 Fig Expansion of lysosomal compartments in the DLM of the *vps24* mutant. Confocal immunofluorescence and native GFP or mCherry fluorescence images of the DLM from WT **(a-d)** or *vps24* mutant **(e-h)** flies exhibiting muscle expression of the lysosomal markers, GFP-LAMP **(A)** or Cathepsin-3xmCherry (CathB-mCh) **(B)**. The *vps24* mutant exhibited accumulation of a ubiquitin-positive lysosomal compartment.
(TIF)

S10 Fig Association of autophagosome-like structures with abnormally enlarged autolysosomes. Transmission electron microscopy images of CNS neurons from the *vps24* mutant. Two representative examples of autophagic intermediates (double arrowheads) closely associated with spherical autolysosome structures. **(A)** is a magnified image of the left bottom corner area in Fig 10Ab.
(TIF)

S1 Table. Numerical data (XLSX)

S1 Raw images (PDF)
