# Supplementary figures and images for: Genetic analysis of the Drosophila ESCRT-III complex protein, VPS24, reveals a novel function in lysosome homeostasis
Source: PLoS One. 2021 May 6;16(5):e0251184. doi: 10.1371/journal.pone.0251184 (PMC8101729; doi:10.1371/journal.pone.0251184)

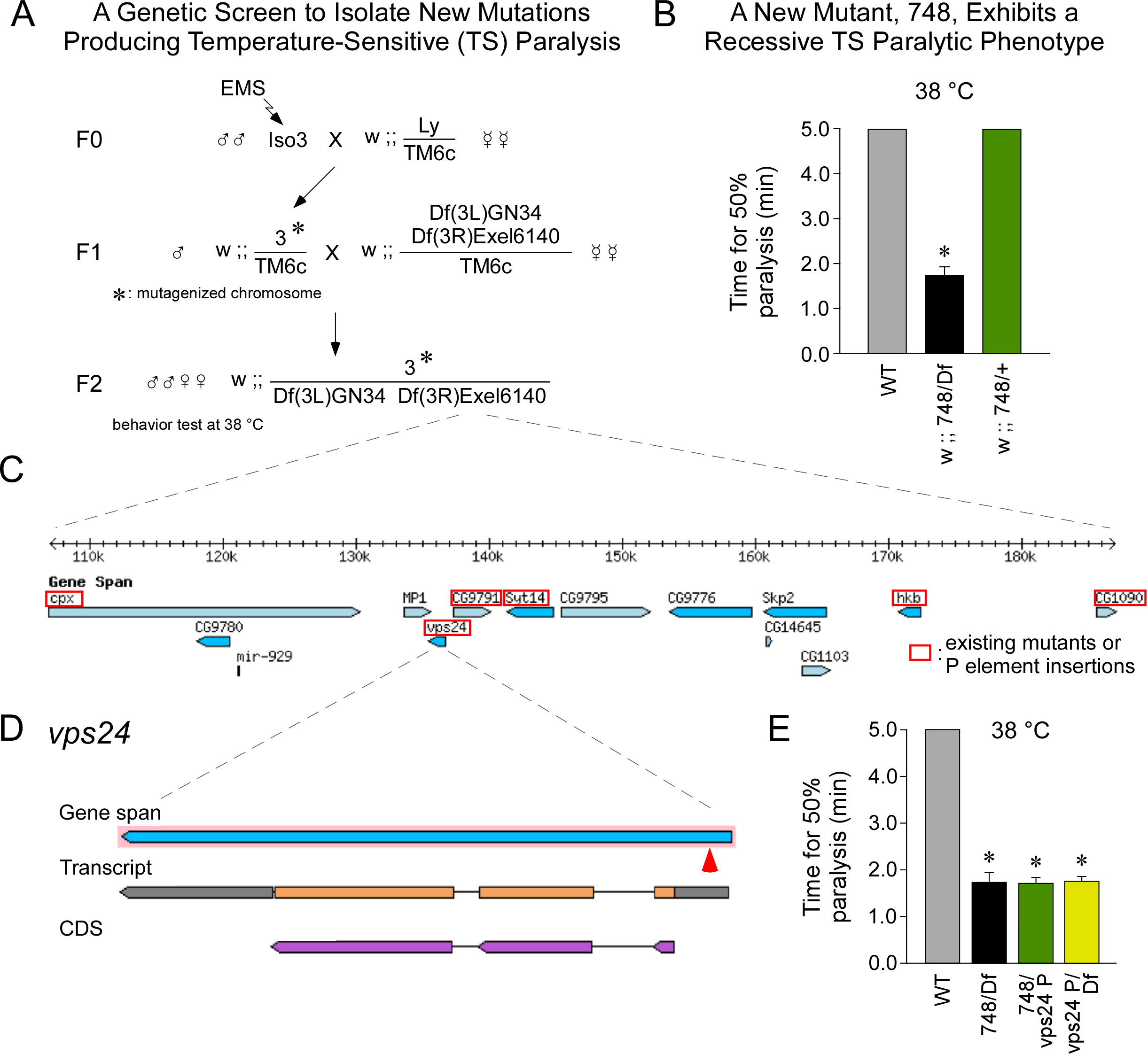

Supplement: S1 Fig — (A) Male flies with an isogenized third chromosome (Iso3) were exposed to ethylmethane sulphonate (EMS). Mutagenized males were mated with females carrying the third chromosome marker, Lyra (Ly), in trans to a balancer chromosome, TM6c. F1 males carrying a mutagenized third chromosome (3*) in trans to TM6c were crossed to females with the third chromosome deficiencies, Df(3L)GN34 and Df(3R)Exel6140. F2 progeny carrying 3* in trans to the deficiency chromosome were screened for motor defects at 38°C. (B) 748/Df(3R)Exel6140 flies (748/Df) exhibited rapid paralysis at 38°C, whereas wild-type flies (WT) did not. Tests of 748/+ flies indicated the phenotype is recessive. Tests were truncated at 5 min if 50% paralysis had not occurred. Here and in subsequent figures, data points represent the mean ± SEM and asterisks mark significant differences from control values (P = 0.05). (C-E) Complementation testing. (C) The deficiency within the right arm, Df(3R)Exel6140, failed to complement 748, whereas Df(3L)GN34 did not. Further complementation testing was performed using six existing mutants that disrupt genes within Df(3R)Exel6140 (boxed in red). (D, E) A P element insertion in the vps24 gene, vps24[EY04708] (red arrowhead in D, vps24 P in E), failed to complement, indicating that the 748 mutation resides in vps24. (TIF) [file pone.0251184.s001.tif]

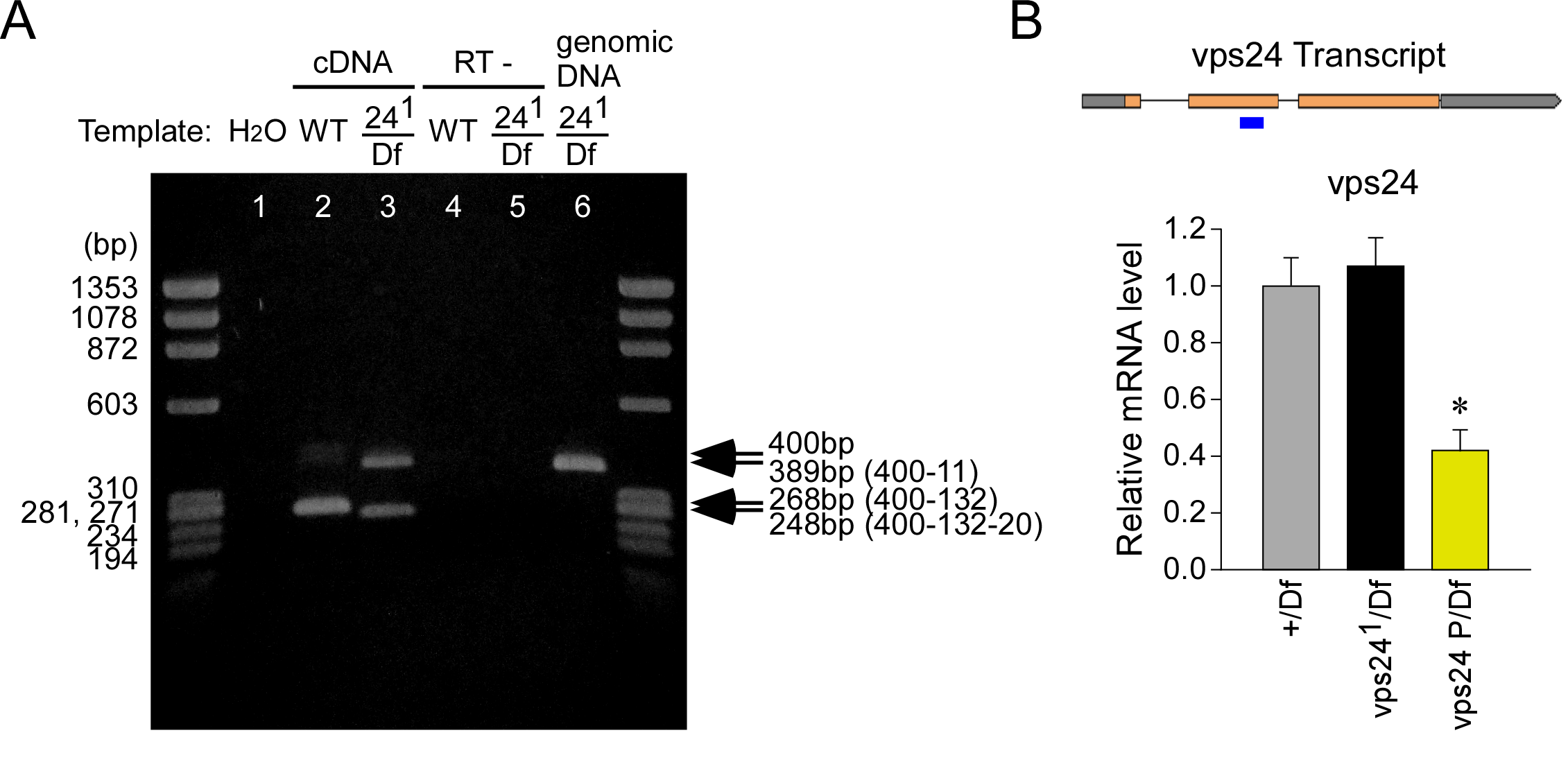

Supplement: S2 Fig — (A) PCR using vps241 cDNA as a template (lane 3) produced 2 kinds of products. The upper band migrated at similar molecular mass to that observed when using the mutant genomic DNA as a template (lane 6). The lower band migrated at a slightly lower molecular mass in comparison to that from wild-type vps24 cDNA (the major band in lane 2). The faint upper band in lane 2 corresponds to the 400 bp PCR product expected from the unspliced vps24 transcript in WT. The 2 kinds of PCR products from the vps241 cDNA were gel-purified and sequenced (see Fig 1D). RT-: cDNA synthesis reaction without reverse transcriptase (RT). (B) Quantitation of vps24 transcript levels by qPCR. The blue bar under the vps24 transcript diagram indicates the location of amplicon examined. The vps24 transcript level of WT control (+/Df) was set at 1.0. The P element insertional allele of vps24 exhibited reduction of the transcript levels by more than 50% relative to WT or vps241. (TIF) [file pone.0251184.s002.tif]

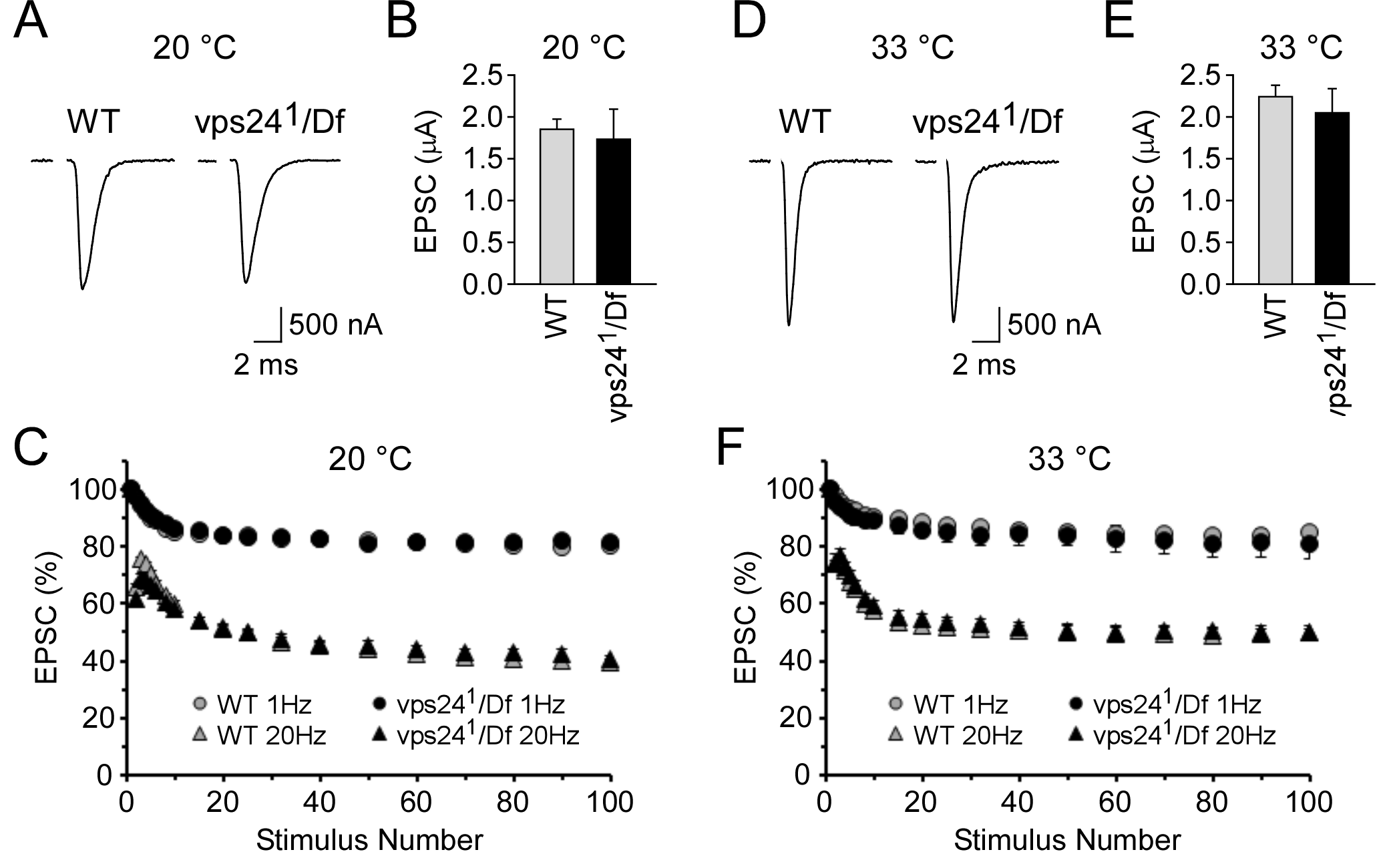

Supplement: S3 Fig — Two-electrode voltage-clamp recordings of excitatory postsynaptic currents (EPSCs) from DLM neuromuscular synapses of WT and the vps24 mutant at permissive (A-C) and elevated (D-F) temperatures. Representative recordings (A, D) and peak amplitude measurements of EPSCs (B, E) indicate wild-type EPSC waveform and amplitude in the vps24 mutant. Stimulation artifacts were removed for clarity. (C, F) The vps24 mutant synapses exhibit wild-type short-term depression during train stimulation at 1 or 20 Hz. Peak EPSC amplitudes were normalized to the initial amplitude and plotted as a function of stimulus number. (TIF) [file pone.0251184.s003.tif]

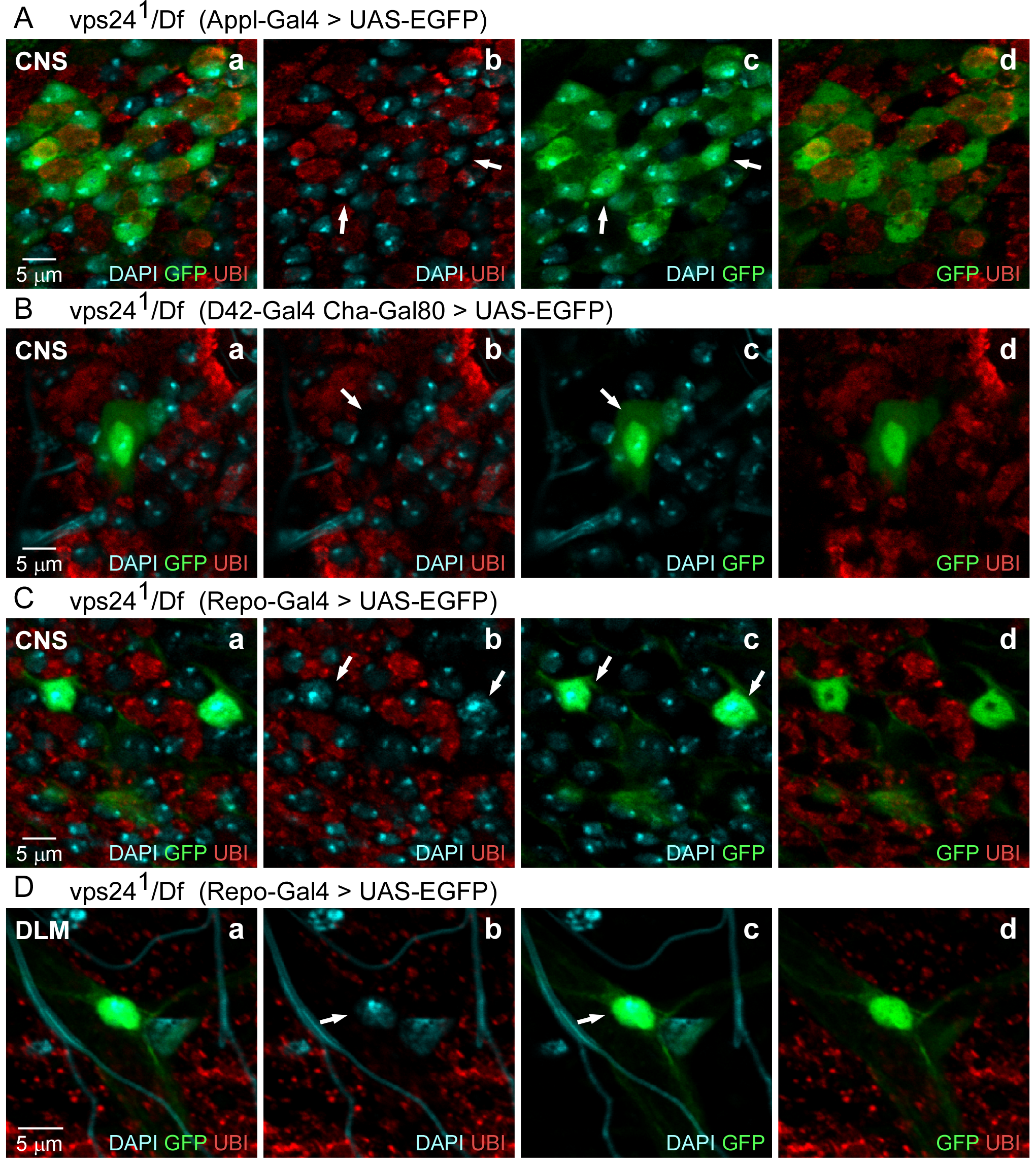

Supplement: S4 Fig — Confocal immunofluorescence and native GFP fluorescence images from the vps24 mutant flies exhibiting neuronal or glial expression of soluble GFP proteins. (A) CNS Neurons labeled by expression of GFP using the Appl-Gal4 neuronal driver. An accumulation of ubiquitin-positive structures was observed in the majority of neurons, though some neurons (arrows) lack these structures. Soluble GFP filled the cytoplasm as well as the nucleus. Note that not all neurons express the transgene. (B) A motor neuron innervating DLM fibers was selectively labeled by expression of GFP using the D42-GAL4 Cha-GAL80 double driver. No ubiquitin-positive structures were observed in the cell body (arrow). (C) CNS glia labeled by expression of GFP using the Repo-Gal4 glial driver. Ubiquitin-positive structures were lacking in CNS glia (arrows). (D) Peripheral Perisynaptic Glia (PPG) labeled by expression of GFP using the Repo-Gal4 glial driver. A cell body of PPG which resides in the periphery over the DLM surface (arrow) lacked ubiquitin-positive structures. (TIF) [file pone.0251184.s004.tif]

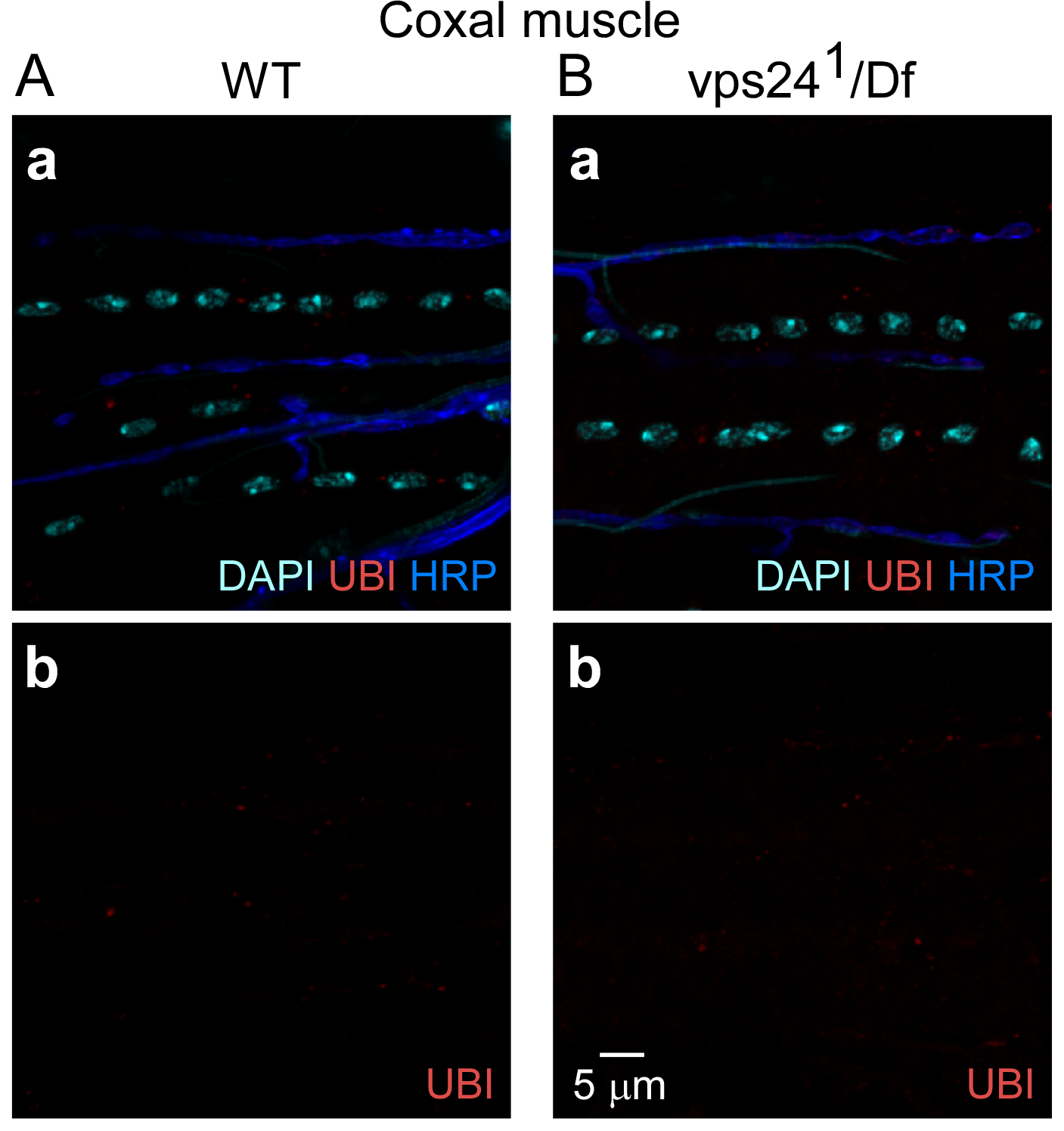

Supplement: S5 Fig — Lack of ubiquitin-positive structures in leg muscle in the vps24 mutant. Confocal immunofluorescence images of leg (coxal) muscle from WT (A) or the vps24 mutant flies (B). The coxal muscles from the vps24 mutant showed no accumulation of ubiquitin-positive structures and resembled WT. (TIF) [file pone.0251184.s005.tif]

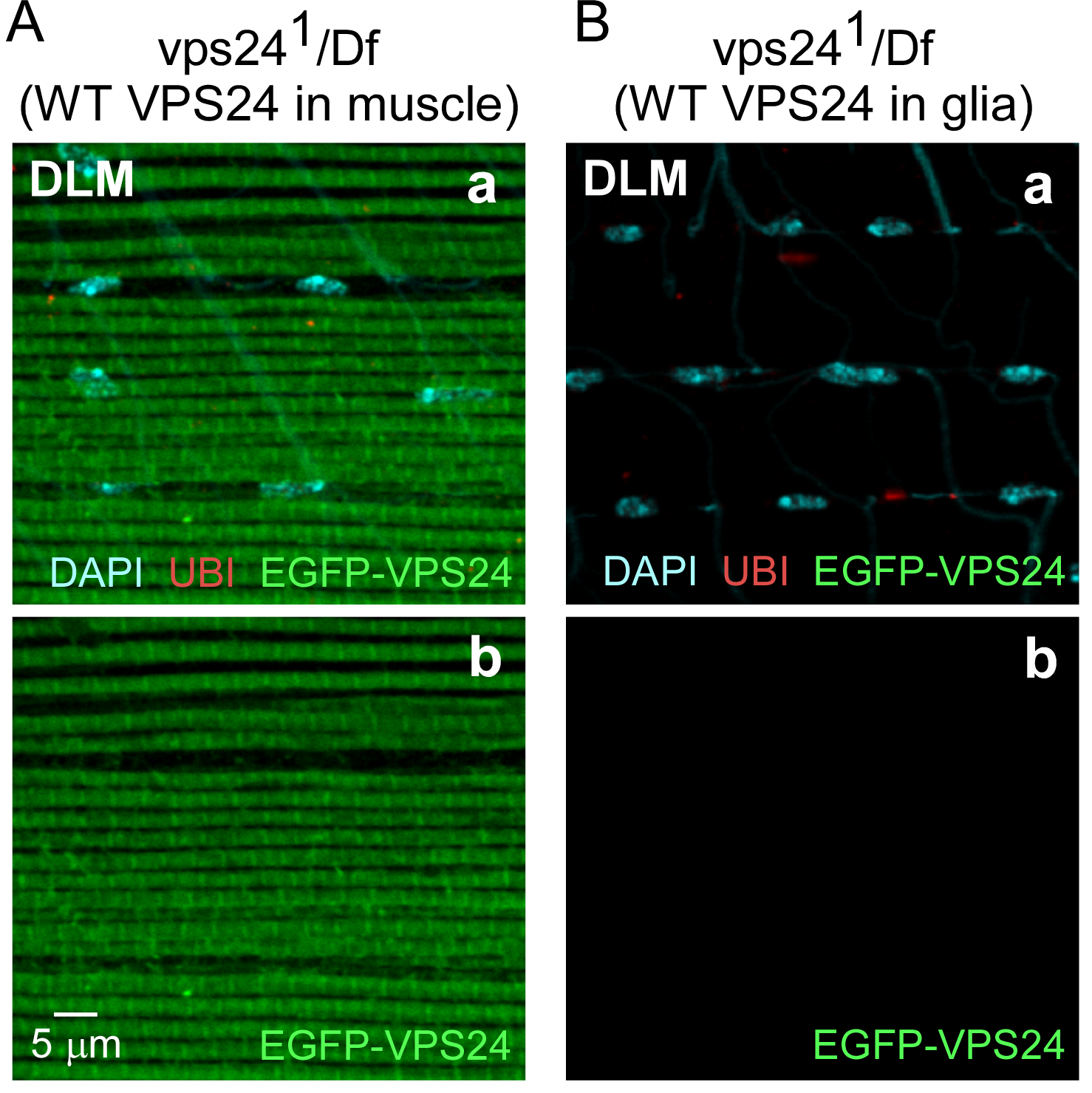

Supplement: S6 Fig — Confocal immunofluorescence and native GFP fluorescence images of DLM from the vps24 mutant flies exhibiting muscle or glial expression of wild-type EGFP-VPS24 protein. (A) Muscle expression of wild-type EGFP-VPS24 produced a clear EGFP-VPS24 signal in the DLM that resembled the muscle contractile apparatus. (B) Glial expression of wild-type EGFP-VPS24 produced no detectable EGFP-VPS24 signal in the DLM but resulted in cell-nonautonomous suppression of the DLM vps24 mutant phenotype. (TIF) [file pone.0251184.s006.tif]

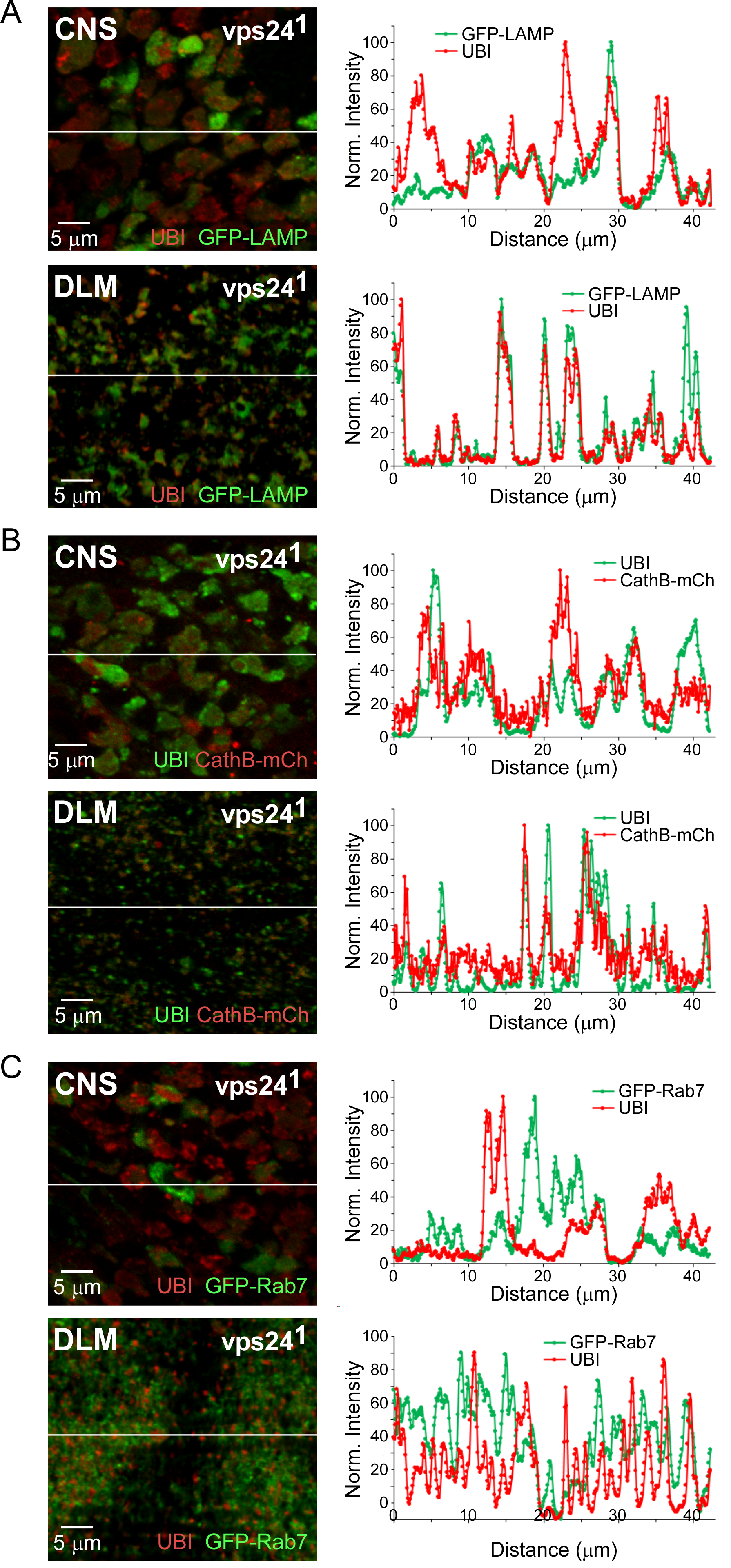

Supplement: S7 Fig — Comparisons of pixel intensity profiles for Ubiquitin with the lysosomal markers (GFP-LAMP) (A) and Cathepsin B (Cathepsin-3xmCherry, CathB-mCh) (B) or the late endosome, autophagosome and lysosome protein, Rab7 (GFP-Rab7) (C) in the vps24 mutant. Images for the CNS correspond to those in Fig 6 and S8A Fig, respectively. Images for the DLM correspond to those in S8B and S9 Figs, respectively. A white line shown in each image designates a line of pixels whose intensities were measured. These values were normalized to the maximum pixel intensity following subtraction of the minimum pixel intensity, and plotted as a function of distance. In both the CNS and DLM, GFP-LAMP and CathB-mCh were colocalized with ubiquitin-positive structures. In contrast, GFP-Rab7 overlaps partially with the ubiquitin-positive lysosomal compartment. (TIF) [file pone.0251184.s007.tif]

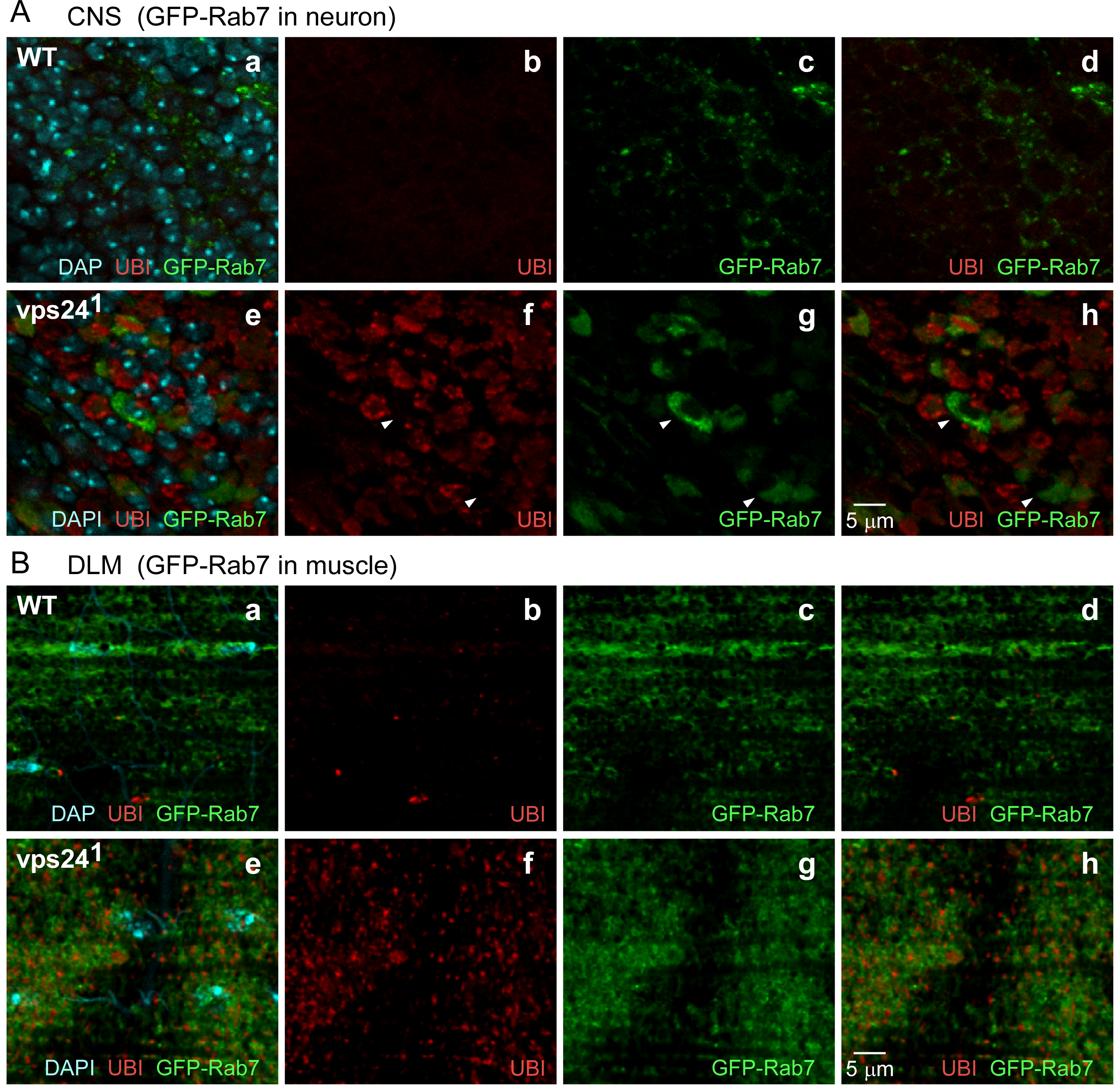

Supplement: S8 Fig — Confocal immunofluorescence images of CNS neurons (A) and DLMs (B) from WT (a-d) or vps24 mutant (e-h) flies exhibiting expression of GFP-Rab7 in the corresponding tissues. (TIF) [file pone.0251184.s008.tif]

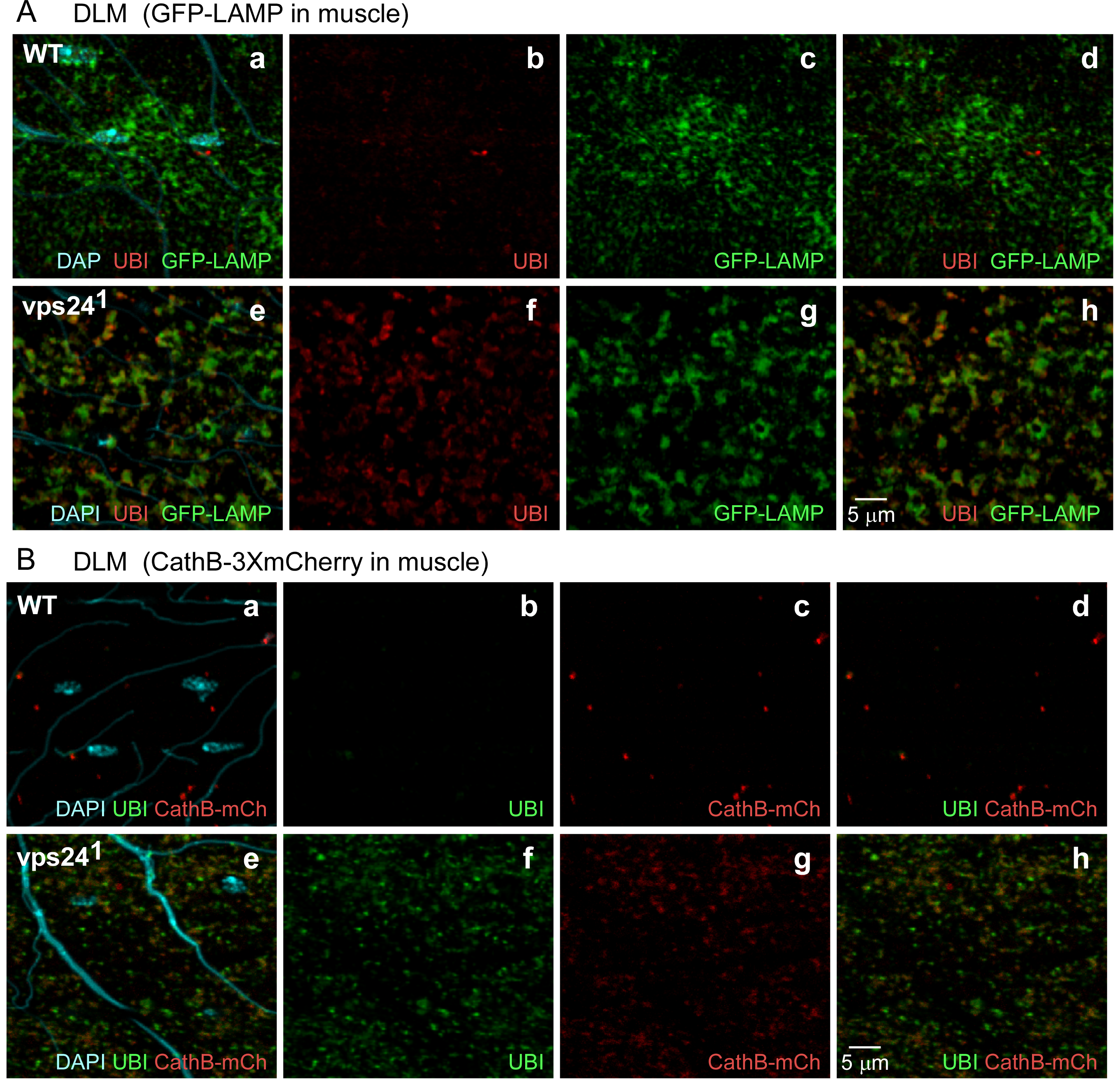

Supplement: S9 Fig — Confocal immunofluorescence and native GFP or mCherry fluorescence images of the DLM from WT (a-d) or vps24 mutant (e-h) flies exhibiting muscle expression of the lysosomal markers, GFP-LAMP (A) or Cathepsin-3xmCherry (CathB-mCh) (B). The vps24 mutant exhibited accumulation of a ubiquitin-positive lysosomal compartment. (TIF) [file pone.0251184.s009.tif]

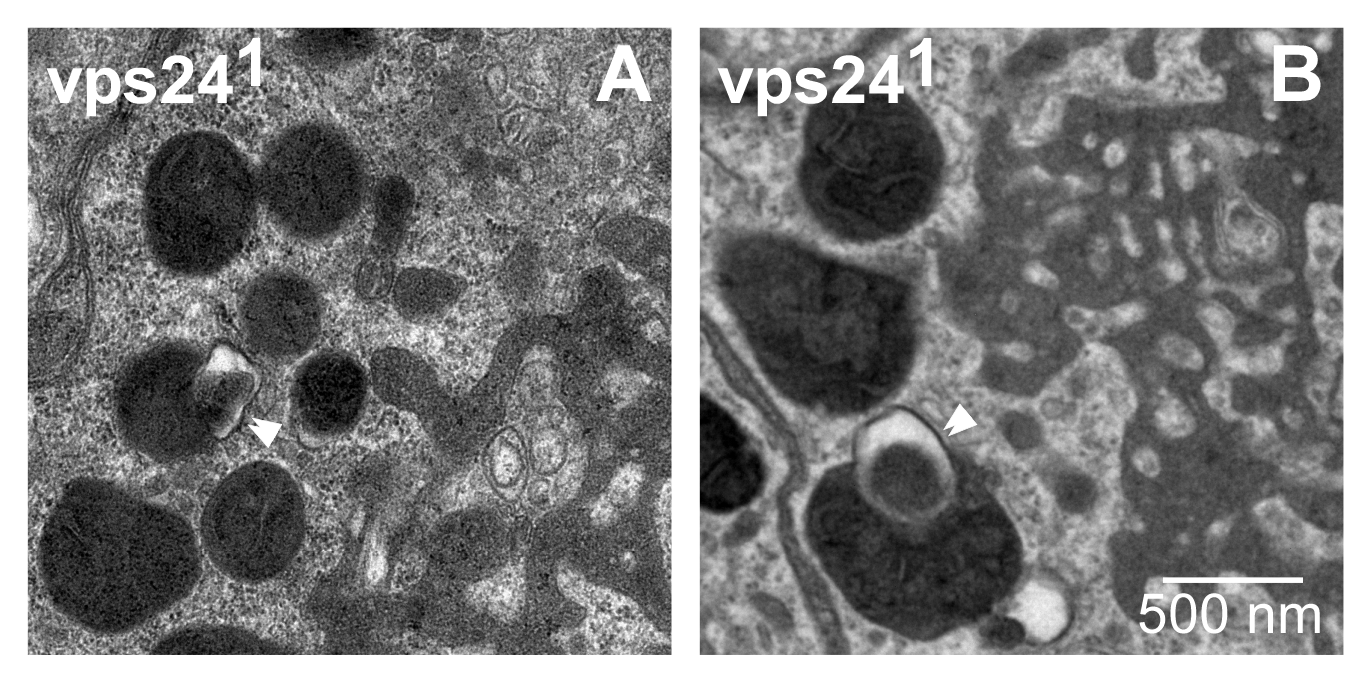

Supplement: S10 Fig — Transmission electron microscopy images of CNS neurons from the vps24 mutant. Two representative examples of autophagic intermediates (double arrowheads) closely associated with spherical autolysosome structures. (A) is a magnified image of the left bottom corner area in Fig 10Ab. (TIF) [file pone.0251184.s010.tif]
