# Supplementary material for: Genetic analysis of the Drosophila ESCRT-III complex protein, VPS24, reveals a novel function in lysosome homeostasis
Source: PLoS One. 2021 May 6;16(5):e0251184. doi: 10.1371/journal.pone.0251184 (PMC8101729; doi:10.1371/journal.pone.0251184)
Supplement: S1 Raw images — (PDF) [file pone.0251184.s012.pdf]

S1_raw_images

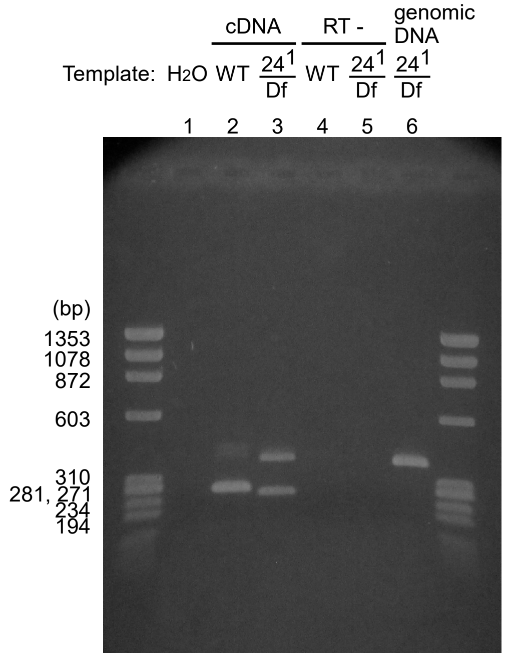

Original image used to generate Figure S2 Panel A
Method used to capture the image: Digital camera through a filter for ethidium bromide gels
Samples: PCR products using indicated materials as a template.
RT- : cDNA synthesis reaction without reverse transcriptase (RT).

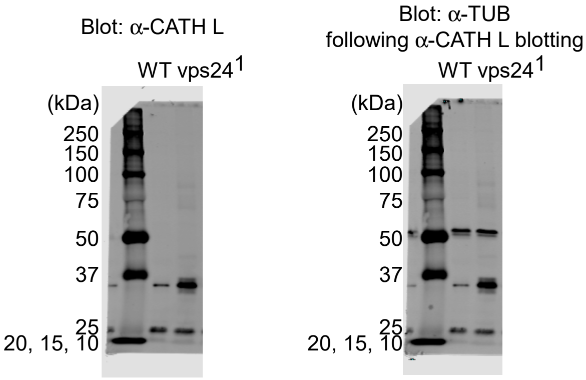

Original images used to generate Figure 7 Panel A
Method used to capture the image: LI-COR OdysseyCLx imager
Samples: Whole fly lysate

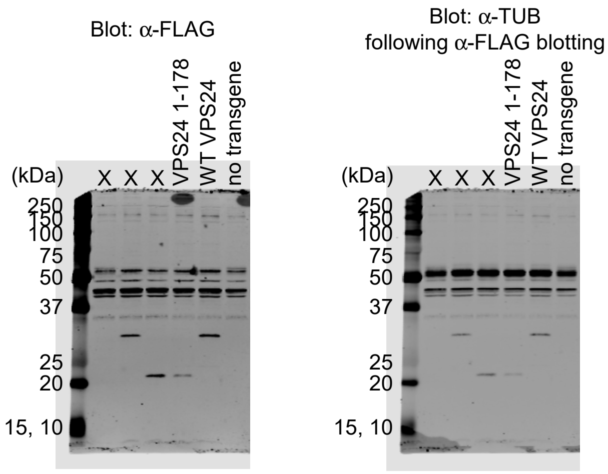

Original images used to generate Figure 11 Panel B
Method used to capture the image: LI-COR OdysseyCLx imager
Samples: Lysate prepared from fly heads
